# WNK1 promotes water homeostasis by acting as a central osmolality sensor for arginine vasopressin release

**DOI:** 10.1172/JCI164222

**Published:** 2023-06-01

**Authors:** Xin Jin, Jian Xie, Chia-Wei Yeh, Jen-Chi Chen, Chih-Jen Cheng, Cheng-Chang Lien, Chou-Long Huang

**Affiliations:** 1 Department of Medicine, Division of Nephrology, University of Iowa Carver College of Medicine, Iowa City, Iowa, USA.; 2 Institute of Neuroscience and; 3 Brain Research Center, National Yang Ming Chiao Tung University, Taipei, Taiwan.

**Keywords:** Endocrinology, Nephrology, Epithelial transport of ions and water, Ion channels, Transport

## Abstract

Maintaining internal osmolality constancy is essential for life. Release of arginine vasopressin (AVP) in response to hyperosmolality is critical. Current hypotheses for osmolality sensors in circumventricular organs (CVOs) of the brain focus on mechanosensitive membrane proteins. The present study demonstrated that intracellular protein kinase WNK1 was involved. Focusing on vascular-organ-of-lamina-terminalis (OVLT) nuclei, we showed that WNK1 kinase was activated by water restriction. Neuron-specific conditional KO (cKO) of *Wnk1* caused polyuria with decreased urine osmolality that persisted in water restriction and blunted water restriction–induced AVP release. *Wnk1* cKO also blunted mannitol-induced AVP release but had no effect on osmotic thirst response. The role of WNK1 in the osmosensory neurons in CVOs was supported by neuronal pathway tracing. Hyperosmolality-induced increases in action potential firing in OVLT neurons was blunted by *Wnk1* deletion or pharmacological WNK inhibitors. Knockdown of Kv3.1 channel in OVLT by shRNA reproduced the phenotypes. Thus, WNK1 in osmosensory neurons in CVOs detects extracellular hypertonicity and mediates the increase in AVP release by activating Kv3.1 and increasing action potential firing from osmosensory neurons.

## Graphical abstract

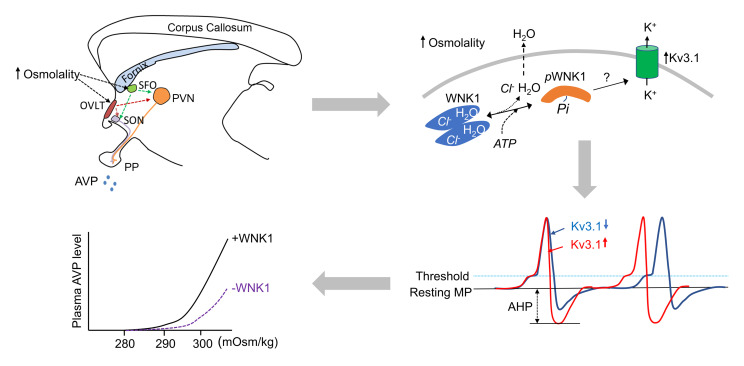

## Introduction

Terrestrial animals are at constant risk of stress due to water deprivation. Maintaining internal osmolality constancy is essential for life. The circumventricular organs (CVOs) of brain, including the organum vasculosum of the lamina terminalis (OVLT; also know as the vascular-organ-of-lamina-terminalis) and subfornical organ (SFO), lack a blood-brain barrier. Neurons in the OVLT and SFO detect increases in serum osmolality (tonicity) and transduce the signals in the form of action potentials (APs) traveling down the axonal process to the magnocellular neurosecretory neurons in the paraventricular nucleus (PVN) and supraoptic nucleus (SON) in hypothalamus (1–3). The median preoptic nucleus inside the blood-brain barrier has reciprocal connections with CVOs and participates in processing and relaying information to other brain regions (1).

Magnocellular neurosecretory neurons in the PVN and SON synthesize antidiuretic hormone arginine vasopressin (AVP), which is transported in vesicles through axonal transport to the posterior pituitary gland for release into blood circulation. APs from CVOs to magnocellular neurons stimulate the release. AVP acts on the kidney to effect free water reabsorption. Hypertonicity also stimulates thirst sensation and drinking with as-yet unknown pathways (4, 5). Together, renal free water reclamation and drinking restore serum osmolality in response to water deprivation. Converse mechanisms defend against hypoosmolality during excessive water drinking. Overall, these feedback regulatory mechanisms maintain osmotic equilibrium near 290 mOsm/kg H_2_O.

The molecular identity of the osmolality sensor(s) in the OVLT and SFO neurons remains elusive. In response to perturbation of the extracellular tonicity, cell volume and thus cell membrane tension change. It has long been postulated that cell membrane–resident proteins, such as mechanosensitive channels, are ideal candidates for detecting changes in extracellular tonicity (6–8). Existing literature on mechanosensitive channels as the central osmolality sensors, however, are inconclusive.

With-no-lysine (WNK) kinases are a family of 4 protein kinases, WNK1–WNK4, with an atypical placement of the catalytic lysine (9–12). Mutations of WNK1 and WNK4 cause an autosomal-dominant hypertension and hyperkalemia (10). WNK4 is heavily expressed in epithelial tissues. In contrast, WNK1 is ubiquitously expressed, including in the central and peripheral nervous system (11, 12). Mutations of WNK1 are known to cause hereditary sensory and autonomic neuropathy type II, a rare autosomal-recessive neurological disorder (13). WNK3 is also expressed in the brain and regulates neuronal intracellular Cl^–^ levels (11). WNKs activate downstream oxidative-stress responsive-1 kinase (OSR1) and related Ste20-related proline/alanine-rich kinase (SPAK) (14). WNKs-OSR1/SPAK kinase cascade regulates ion channels and transporters, including many types of K^+^ channels, epithelial Na^+^ channel ENaC, and cation-chloride cotransporters NKCC1, NKCC2, and NCC. In cultured cells, WNK kinase activity is activated by extracellular hyperosmolality (11, 14, 15).

In many cells, cell volume recovers following hypertonicity-induced shrinkage via the process of regulatory volume increase (RVI). Activation of NKCC1 via WNKs-OSR1/SPAK cascade plays an important role in RVI (11, 14, 15). However, the experimental-induced activation of WNKs-OSR1-NKCC1 and cell volume increase has been studied using a rather large hypertonic challenge, approximately 100 mOsm/kg above baseline. While this degree of osmotic challenge occurs in the kidney medulla, the relevance of osmotic stimulation of WNK kinase cascade to renal fluid and electrolyte transport is unclear. There are no physiological conditions in which hypertonicity stimulates WNKs to effect transepithelial salt transport in the kidney. Moreover, in transporting renal epithelia, ion fluxes across one membrane (e.g., basolateral) will be tightly coupled by parallel entry on the other membrane (e.g., apical). The tight apical-basolateral transport coupling is to minimize fluctuations of intracellular concentration of solutes and cell volume while moving a large quantity of ions across the epithelia. Overall, the role of WNKs in osmoregulation in the whole animal is unknown.

Here, we showed that WNK1 functions as a central osmolality sensor in vivo that detects physiological ranges of hypertonicity to stimulate AVP release. Loss of this function of WNK1 in the brain leads to defects in AVP release and in water homeostasis. Our findings reveal that an intracellular protein acts as a sensor for extracellular tonicity and provide fresh insights into the mechanism of how the body maintains osmolality constancy.

## Results

### Mice with neuron-specific deletion of Wnk1 have polyuria and relative hypotonic urine compared with WT mice.

**Figure 1:**
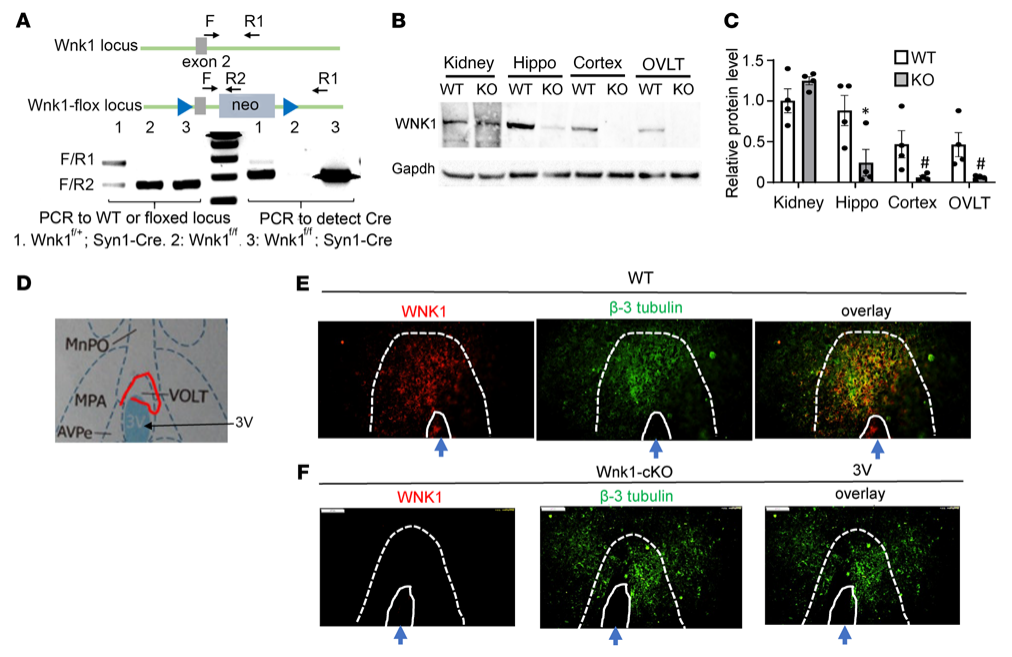
Neuron-specific conditional KO of *Wnk1* markedly reduces WNK1 in brain regions, including OVLT. (**A**) Genotyping of *Wnk1*-cKO mice mediated by neuron-specific *Syn1-Cre*. Genomic tail-clip DNA was used for analysis. Lane 1, *Wnk1^fl/+^*;*Syn1-Cre*; lane 2, *Wnk1^fl/fl^*; lane 3, *Wnk1^fl/fl^*;*Syn1-Cre*. PCR is shown to detect WT vs. *Wnk1^fl/fl^* locus (exon 2 and neo cassette are floxed). PCR forward primer F is located at exon 2. Reverse primers R1 and R2 are located at intron 2 and neo cassette, respectively. Note that *Syn1-Cre* is only active in neurons, so unexcised *Wnk1^fl/fl^* locus is detected in tail-clip DNA. With large size neo cassette in the floxed locus, the F/R1 primer set does not amplify under the condition of PCR reaction. Additionally, PCR is shown to detect *Syn1-Cre* using Cre-specific primers. (**B**) Representative Western blot of WNK1 protein in WT and cKO brain regions shown relative to the kidney. Hippo, hippocampus. Cortex, cerebral cortex. (**C**) Quantitation (mean ± SEM) of 4 separate experiments, as shown in **B**. One WT and cKO mouse for each experiment. WNK1 was normalized to Gapdh and compared with the WT kidney (set as “1”). *P < 0.05, ^#^*P* < 0.01, KO vs. WT by unpaired *t* test. (**D**) Atlas of brain section for immunofluorescent staining, as in **E** and **F**. OVLT (also known as vascular-organ-of-lamina-terminalis [VOLT]) is marked by a red line. 3V, third ventricle; MnPO, median preoptic nucleus; MPA, medial preoptic area. (**E** and **F**) Immunofluorescent staining of WNK1 in OVLT neurons colocalized with neuronal marker β-3 tubulin in WT (**E**) and cKO (**F**) mice. Scale bar: 100 μm.

WNK1 kinase is ubiquitously expressed. Mice with global deletion of the *Wnk1* gene are embryonic lethal due to angiogenesis defects (16, 17). We have generated viable adult mice with global *Wnk1* deletion rescued by expression of constitutive-active OSR1 in endothelia but not in the kidney and brain. We found that these mice had relatively higher urine output and lower urine osmolality compared with WT littermates, which persists during water restriction (data not shown), suggesting that these mice have diabetes insipidus (DI). To investigate whether the defects are due to loss of WNK1 in the central nervous system or the kidney (i.e., central vs. nephrogenic DI), we generated mice with neuron-specific conditional KO (cKO) of *Wnk1* using synapsin1-Cre (*Wnk1^fl/fl^*; syn1-Cre) (Figure 1A). In normal WT mice, WNK1 protein abundance in brain regions including the OVLT was lower than in the kidney, yet with significant expression (Figure 1, B and C). In neuronal specific *Wnk1*-cKO mice, WNK1 was markedly reduced in brain regions but not in the kidney. Immunofluorescent staining confirmed WNK1 expression in neurons in brain regions including the OVLT, SFO, and cerebral cortex of WT mice, and conditional deletion of *Wnk1* markedly reduced the expression (Figure 1, D–F, and Supplemental Figure 1; supplemental material available online with this article; https://doi.org/10.1172/JCI164222DS1).

**Figure 2:**
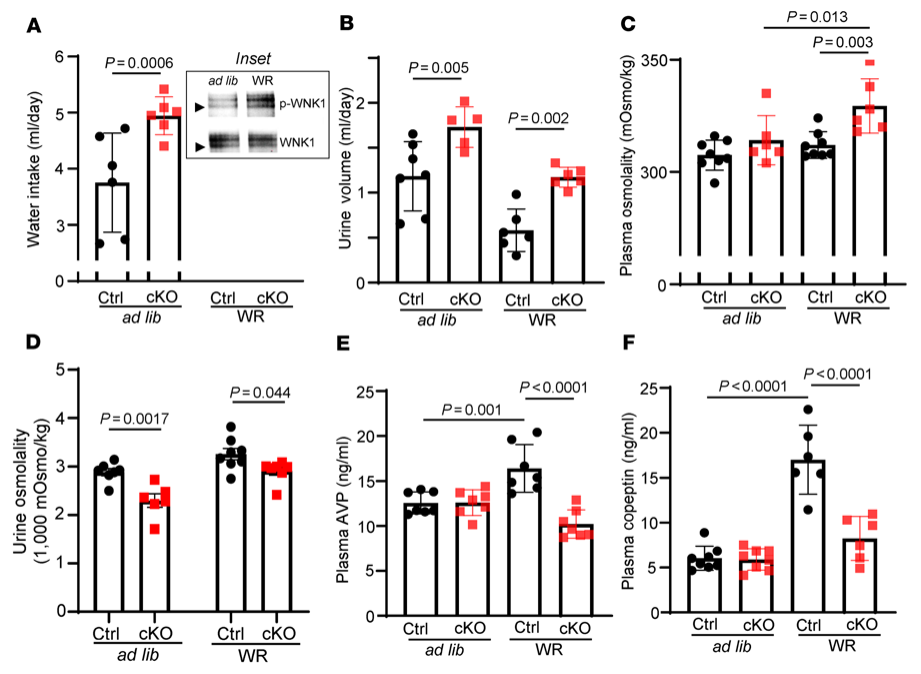
*Wnk1*-cKO mice exhibit partial central diabetes insipidus with impaired AVP and copeptin release in response to water restriction. (**A**) Water intake, (**B**) urine volume, (**C**) plasma osmolality, (**D**) urine osmolality, (**E**) plasma AVP level, and (**F**) copeptin level of control (Ctrl) and cKO mice at either ad libitum water intake or after 24-hour water restriction (WR). The inset in **A** shows Western blotting analysis of abundance of total and phospho-WNK1 (p-WNK1) using antibody against total WNK1 and against S382 phospho-WNK1. Arrowheads indicate molecular size 250 kDa. Lysates from WT OVLT tissue at ad libitum water intake and after 24-hour water restriction were immunoprecipitated by anti-WNK1 antibody and probed by anti-WNK1 and anti-p-WNK1 antibody. Representative of 4 separate experiments. Each experiment consists of 1 mouse ad libitum and 1 mouse on water restriction. For statistical analysis was performed with 2-way repeated ANOVA with Šidák post hoc analysis; for statistical analysis of the inset in **A**, unpaired 2-tailed *t* test was performed. For bar graphs, *n* = 6–8 mice, as indicated in scatter plots.

**Figure 3:**
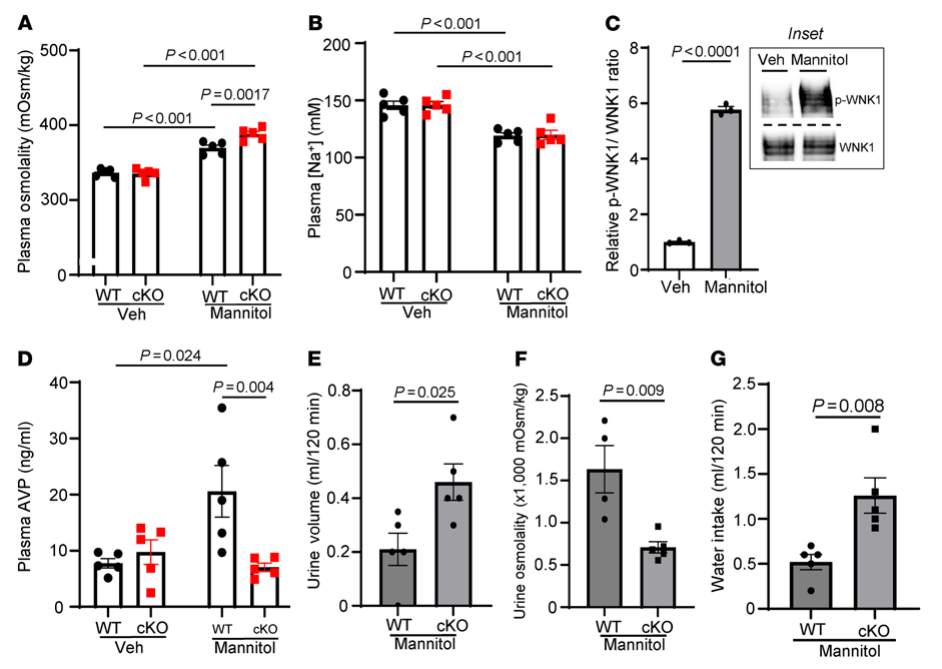
*Wnk1* deletion impairs hyperosmolality-induced AVP release but not osmotic thirst. (**A**) Plasma osmolality, (**B**) [Na^+^], (**C**) relative p-WNK1/WNK1 ratio in OVLT, (**D**) plasma AVP, (**E**) urine volume, (**F**) urine osmolality, and (**G**) water intake in WT and *Wnk1*-cKO mice after mannitol or vehicle injection. Urine volume and water intake were measured 120 minutes after injection. Other measurements were taken 30 minutes after injection in separate mice from those in which urine and water intake were measured. The inset in **C** is representative of 3 experiments. Each experiment consists of 1 mouse injected with vehicle and 1 mouse injected with mannitol. Statistical analysis in **A**, **B**, and **D** was performed with 2-way repeated ANOVA with Šidák post hoc analysis; otherwise, unpaired *t* test was used. For bar graphs in **A**, **B**, **D**–**G**, *n* = 5 mice for each experimental condition, as indicated in scatter plot.

Autophosphorylation of WNK1 at S382 within the kinase domain reflects WNK1 kinase activation (9, 18–20). We found that water restriction (WR) increased the abundance of S382 phospho-WNK1 (Figure 2A, inset, and Supplemental Figure 2), supporting the role of WNK1 as an osmosensor. Balance studies revealed that water intake and urine output were significantly higher in cKO mice compared with control littermates (control; *Wnk1^fl/fl^* or *Wnk1^fl/+^* without Cre) under free access to water (ad libitum) (Figure 2, A and B). While plasma osmolality was not significantly different during ad libitum, urine osmolality was lower in *Wnk1*-deleted mice compared with control mice (Figure 2, C and D). Polyuria with relative urine hypotonicity in *Wnk1*-cKO mice versus control mice persisted during water restriction (Figure 2, B and D). Plasma osmolality became significantly higher in cKO mice compared with control mice during water restriction (Figure 2C). These findings that polyuria and relative hypotonic urine persist in water restriction indicate DI, not polydipsia, as the underlying cause. Higher water intake in cKO mice is a compensatory response to polyuria (see Figure 3 below for effect of *Wnk1* deletion on osmolality-induced thirst).

### Neuronal deletion of Wnk1 impairs hypertonicity-stimulated release of AVP.

To further support the central DI phenotypes of neuronal *Wnk1* deletion, plasma levels of AVP and copeptin were measured. The half-life of circulating endogenous AVP is several minutes. Copeptin is the inactive N-terminal fragment of pre-pro-AVP, which is more stable in circulation and believed be a more reliable measurement of AVP release. As shown, basal levels of AVP and copeptin were not significantly different between cKO and control mice (Figure 2, E and F). Water restriction stimulated release of AVP and copeptin in control mice, and the increases were blunted in *Wnk1*-cKO mice. Thus, WNK1 is involved in hypertonicity-induced AVP release. Additional molecules or pathways besides WNK1 may be involved in regulating AVP release at least at the basal state (see Discussion and below). Overall, the central DI phenotypes of *Wnk1*-cKO mice is partial. With ad libitum water access, cKO mice appear grossly normal with indistinguishable activity level and apparent normal growth curve and body size compared with control mice (data not shown).

### Neuronal deletion of Wnk1 does not impair hypertonicity-stimulated thirst.

Hypertonicity stimulates thirst as well as release of AVP. Whether the two processes are mediated by the same molecular mechanism is unknown (21). The finding that cKO mice have higher water intake (than control mice) to compensate for polyuria (Figure 2A) suggests that *Wnk1* deletion does not affect hypertonicity-induced thirst. Here, we used intraperitoneal mannitol injection to further examine the role of WNK1 kinase in osmolality-induced AVP release and thirst response. Mannitol injection raised plasma osmolality in both WT and cKO mice in 30 minutes (Figure 3A). The increases were significantly higher in cKO mice compared with WT mice (see below). Plasma [Na^+^] was decreased in mannitol-injected mice due to dilution by water extraction from cells (Figure 3B). Hypertonicity induced WNK1 phosphorylation in OVLT neurons in 30 minutes (Figure 3C) and increased plasma AVP levels in WT but not in *Wnk1*-cKO mice (Figure 3D). Osmolality and water load from mannitol injection induced significant urine output within 120 minutes. Consistent with the finding that *Wnk1* deletion impairs AVP release, mannitol-induced urine volume was significantly higher and urine osmolality lower in cKO mice compared with that in WT mice (Figure 3, E and F). The relative hypotonic urine also explains higher plasma osmolality in cKO mice (Figure 3A). Water intake in response to hypertonicity was significantly higher in cKO mice than in WT mice (Figure 3G). Polyuria and hypotonic urine caused by defective AVP release likely account for higher plasma osmolality and thus higher water intake in cKO versus WT mice. Overall, these results using mannitol injection agree with the results shown in Figure 2 by using water restriction to increase plasma tonicity. They support the notion that *Wnk1* deletion impairs AVP release but not osmolality-induced thirst.

### Hypertonicity stimulates K^+^ current-mediated membrane potential oscillation in OVLT neurons involving WNK1 kinase.

**Figure 4:**
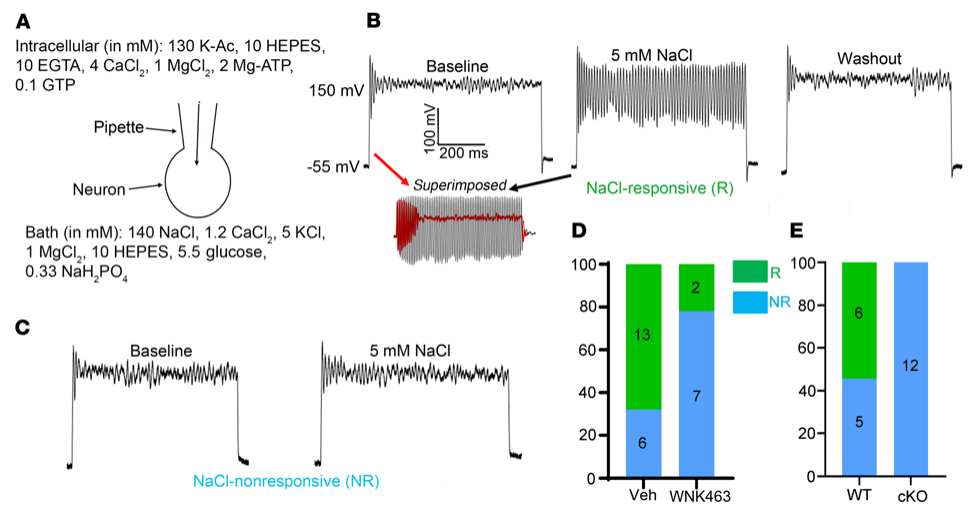
Hypertonicity induces membrane potential oscillation in freshly isolated OVLT neurons mediated by WNK1. (**A**) Ruptured whole-cell current-clamp recording for membrane potentials. Pipette and bath solution are indicated. (**B** and **C**) Membrane potentials of freshly isolated OVLT neurons at baseline, after incubation with 5 mM NaCl for 3 minutes and 5 minutes after washout of 5 mM NaCl hypertonicity. 600 pA currents were injected to depolarize membrane potential from the resting potential –55 mV to +150 mV. **B** and **C** represent examples of NaCl-responsive and nonresponsive neurons, respectively. (**D**) Treatment with pan-WNK kinase inhibitor (WNK463). Green and cyan bars indicate responsive (R) and nonresponsive (NR), respectively. WNK463 treatment significantly decreased the percentage distribution of responsive neurons vs. vehicle (Veh) treatment. *P* < 0.01, WNK463 vs. Veh, by 2-tailed Fisher’s exact test. (**E**) *Wnk1*-cKO eliminated NaCl responsiveness. *P* < 0.01, cKO vs. WT, by 2-tailed Fisher’s exact test. In **D** and **E**, OVLT neurons were isolated form 4–5 mice for vehicle-treated, WNK463-treated, WT, and cKO groups.

To further examined the role of WNK1 kinase in osmosensory neurons, we isolated OVLT neurons for whole-cell current-clamp recording. Current-clamp recording was performed with bath containing 140 mM NaCl and 5 mM KCl; pipette containing 130 mM K-acetate; and injection of 600 pA currents over 500 milliseconds (Figure 4A). Injecting 600 pA currents depolarized freshly isolated OVLT neurons to approximately +150 mV and elicited membrane potential oscillation that decayed rapidly in the baseline condition (Figure 4B). Recording from the same neuron after incubation with an additional 5 mM NaCl (i.e., bath contains 145 NaCl) for 3–5 minutes resulted in marked enhancement in membrane potential oscillation (Figure 4B). Superimposition further illustrated the difference between baseline and 5 mM NaCl. Rerecording after washout of 5 mM NaCl revealed that membrane potentials were restored to the baseline without oscillation (Figure 4B). As previously reported (22, 23), OVLT neurons are heterogeneous, not every neuron responded to hypertonic challenge (Figure 4C represents an example of a nonresponsive neuron). The percentage of NaCl-responsive neurons was used as a readout. As shown, in the control condition (labeled vehicle, i.e., DMSO), 13 of 19 neurons recorded (68%) exhibited hypertonicity-induced membrane potential oscillation (Figure 4D). In contrast, preincubation of isolated neurons with a pan-WNK inhibitor, WNK463 (10 μM for 3 hours) (24), significantly reduced the percentage of neurons (22%, 2 of 9) that responded with oscillation. In further support for the role of WNK1, hypertonicity-induced membrane potential oscillation was impaired in neurons isolated from neuron-specific *Wnk1*-cKO mice (Figure 4E). We also found that 10 mM mannitol (on top of 140 NaCl in bath) exerted the same effect (Supplemental Figure 3), indicating that it is activated by osmolality, not selectively by Na^+^. Thus, hypertonicity activates membrane channels in OVLT neurons through WNK1 cascade.

Release of AVP is also stimulated by hypovolemia through peripheral baroreceptor (25, 26). Hypovolemia also stimulates thirst through angiotensin II generated centrally and peripherally (27, 28). Whether baroreceptor-mediated AVP release and angiotensin II–stimulated thirst converge on same osmosensory neurons that utilize WNK1 as the molecular sensor is unknown. The 24-hour water restriction employed in our experiment increases plasma osmolality by approximately 2%–3% (~8 mOsm/kg increase on baseline 310 mOsm/kg, Figure 2C). An estimated more than 8%–10% reduction in central volume is required to stimulate AVP and angiotensin II release (25–28). Thus, baroreceptor-mediated AVP release is not likely involved in our experiment. Nonetheless, we asked whether angiotensin II could activate membrane potential oscillation in isolated OVLT neurons and found that it did not whereas the positive control 5 mM NaCl did (Supplemental Figure 4).

We further characterized the biophysical basis of membrane potential oscillation. OVLT neurons contain voltage-activated Na^+^ and K^+^ channels, mediating the membrane depolarization and repolarization phase of an AP, respectively. The membrane oscillation that we observed here was not an AP, as the repolarization did not reach below threshold membrane potential. The threshold membrane potential for opening Kv channels is more positive to the equilibrium potential for K^+^ (E_K_). Thus, we reasoned that the membrane potential oscillation is due to membrane potential alternating between depolarization from injecting positive currents and hyperpolarization from K^+^ efflux passing through open voltage-activated Kv channels. To validate the hypothesis, we replaced pipette and bath K^+^ with nonpermeant Cs^+^ and found that it eliminated membrane potential oscillation (Supplemental Figure 5A). The high activation threshold and delayed inactivation kinetics suggest that high-threshold Kv channels, such as Kv2 and Kv3 channels, are involved (29, 30). We then used tetraethylammonium (TEA) to distinguish between Kv2 and Kv3 channels. While all K^+^ channels are susceptible to blockade by millimolar concentrations of TEA applied intracellularly, extracellular TEA selectively blocks Kv3 channels with IC_50_ of approximately 300 μM (30). We found that 3 mM TEA completely blocked hypertonicity-induced responses in OVLT neurons (Supplemental Figure 5B), supporting that it is due to K^+^ efflux through Kv3 channels (see below for further identification of Kv3’s channel).

### Kinase activity of WNK1 is involved in hypertonicity stimulation of K^+^ currents in OVLT neurons.

**Figure 5:**
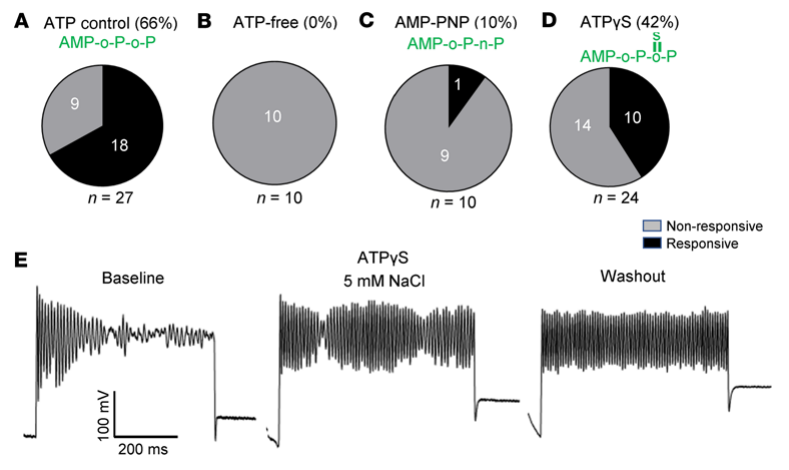
Effects of removal of intracellular ATP or substitution by ATP analogs on hypertonicity-induced membrane potential oscillation. Whole-cell patch-clamp recordings were performed as in Figure 3, with the exception that ATP in the pipette was removed or replaced as indicated. [Mg^2+^] was kept constant. (**A**) Control experiments with 2 mM ATP in the patch pipette. (**B**) Zero ATP in the patch pipette. (**C**) Patch pipette contained 2 mM AMP-PNP. (**D**) Patch pipette contained 2 mM ATPγS. (**E**) With ATPγS in the pipette, in 7 of 10 cells that responded to hypertonicity stimulation, membrane potential oscillation persisted after hypertonic NaCl was washed out. Shown is a representative example of persistent oscillation after washout. Note that ATPγS is a substrate for kinase but not for phosphatase due to thio-linkage between sulfur and oxygen atom. Pie charts in **A**–**D** show distribution of responsive and nonresponsive neurons. **B** and **C** are statistically significantly different from **A**, *P* < 0.05 by 2-tailed Fisher’s exact test. OVLT neurons were isolated from 4–6 mice for each experimental setting.

Additional support for the notion that kinase activity of WNK1 is involved comes from the following results of studies substituting Mg-ATP in the pipette with ATP-free solution or ATP analogs. As shown in neurons recorded with pipette containing 2 mM Mg-ATP, 66% of neurons responded to 5 mM NaCl hypertonic challenge (Figure 5A). In ATP-free pipette solution, 0% of neurons responded (Figure 5B). When substituted with the nonhydrolyzable AMP-PNP, only 10% of neurons responded (Figure 5C). Incomplete suppression by AMP-PNP is likely due to residual ATP in the pipette. ATPγS is an ATP analog that functions as a substrate for protein kinase but not for protein phosphatase (31). Interestingly, when substituted with ATPγS, 42% of neurons responded to hypertonic challenge (Figure 5D). Among the 10 neurons that responded, 7 of 10 did not exhibit washout (Figure 5E shows an example without washout). Thus, hypertonicity-induced Kv3-mediated membrane potential oscillation response is due to ATP- and protein kinase–mediated phosphorylation. Dephosphorylation by protein phosphatase(s) underlies the fast recovery when hypertonic stimulation is removed. Overall, these results strongly support the hypothesis that phosphorylation by WNK1 kinase and dephosphorylation by a phosphatase(s) are important for the on/off effect of detecting hypertonic stress.

### WNK1 in PVN-projecting CVO neurons is involved in hyperosmolality regulation of AVP release and water homeostasis.

**Figure 6:**
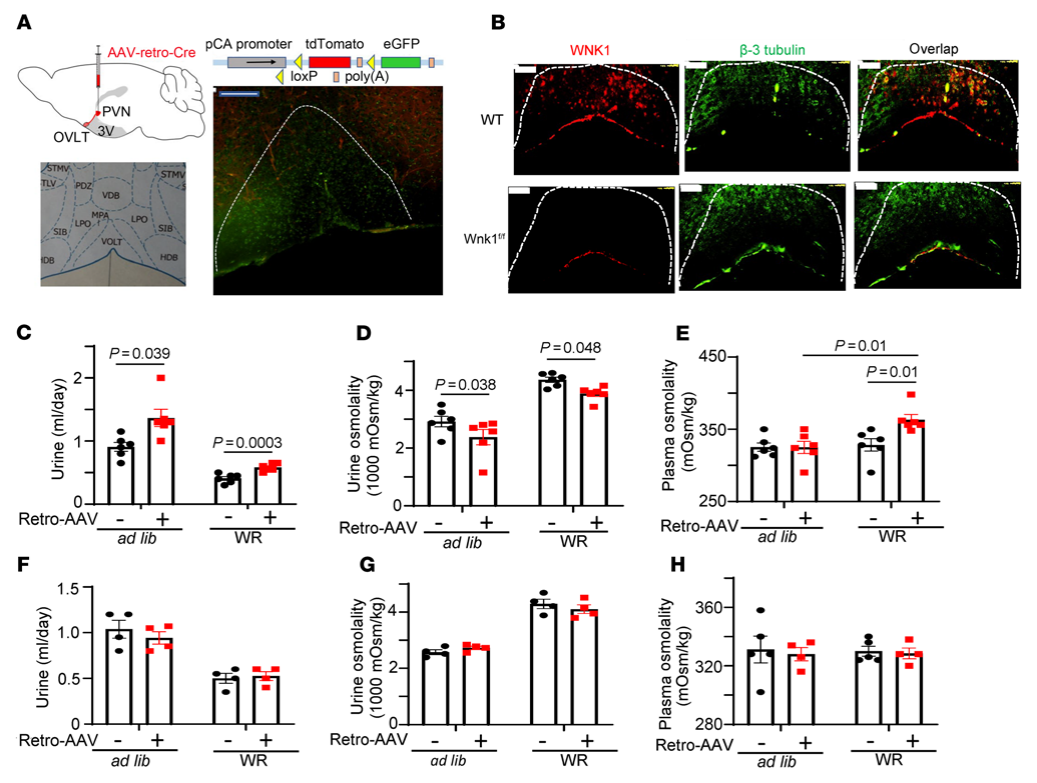
Deletion of *Wnk1* in PVN-projecting OVLT neurons is responsible for the partial CDI phenotype. (**A**) Injection of AAV-retro-Cre virus into PVN of tdTomato-EGFP reporter mice resulted in green fluorescence in neurons of OVLT nuclei, which otherwise exhibited tomato red fluorescence. Scale bar: 200 μm. (**B**) PVN injection of AAV-retro-Cre virus into *Wnk1^fl/fl^* mice resulted in deletion of *Wnk1* in OVLT compared with control experiments with injection of AAV-retro-Cre virus into PVN of WT mice. Scale bar: 100 μm. (**C**) Urine volume, (**D**) urine osmolality, and (**E**) plasma osmolality of *Wnk1^fl/fl^* mice before and after injection with AAV-retro-Cre virus during at libitum and after water restriction (WR). (**F**) Urine volume, (**G**) urine osmolality, and (**H**) plasma osmolality of WT mice before and after injection with AAV-retro-Cre virus. Data shown are mean ± SEM from before injection (labeled retro-AAV –) and after injection (labeled retro-AAV +). Statistical analysis by 2-way repeated ANOVA with Šidák post hoc analysis. *n* = 4–6 mice as indicated by scatter plots.

Osmosensory neurons in CVOs project to AVP-producing magnocellular neurosecretory neurons in the PVN and SON. Here, we used retrograde neuronal tracing to further investigate the hypothesis that WNK1 in CVO neurons is involved with osmolality detection. Subsets of OVLT neurons (Figure 6A) and SFO neurons (data not shown) displayed strong EGFP fluorescence (rather than tdTomato red fluorescence at baseline) following injection of retrograde adeno-associated virus–Cre recombinase (AAVrg-Cre) into the PVN of tdTomato-EGFP (mT/mG) reporter mice. The results validate the approach to reach CVO neurons by injecting retrograde AAV into the PVN.

Next, we studied *Wnk1^fl/fl^* mice in which the PVN was injected with retrograde AAVrg-Cre to delete *Wnk1* in CVOs. WT mice were used as controls; they were injected with PVN with the same retrograde AAVrg-Cre. Immunofluorescent staining confirmed that WNK1 was markedly reduced in OVLT neurons in *Wnk1^fl/fl^* mice (but not in WT mice) in which the PVN was injected the retrograde AAVrg-Cre (Figure 6B). Water intake (data not shown), urine output, and urine osmolality were measured before and after retrograde AAV-Cre injection during ad libitum water access and water restriction. Supplemental Figure 6 shows the experimental protocol and timeline. Figure 6, C–E, show results from experiments with *Wnk1^fl/fl^* mice; Figure 6, F–H, shows result from experiments with control mice. In experimental mice with ad libitum water access, 10 days after injection urine output was significantly increased and urine osmolality significantly decreased versus before injection (Figure 6, C and D). Water restriction studies were performed to determine whether water homeostasis defects in mice received AAVrg-Cre injection in the PVN are due to DI or untoward effect of polydipsia from PVN injection. Polyuria and relative hypotonic urine persisted during water restriction (Figure 6, C and D), indicating that the effect is due to DI. Plasma osmolality was not different before and after retrograde AAVrg at ad libitum, but the difference became apparent with water restriction (Figure 6E). For control mice, there were no differences in these parameters before and after injection (Figure 6, F–H). The effect was due to deletion of *Wnk1* in CVOs not in the PVN, as injecting nonretrograde AAV-Cre into the PVNs of *Wnk1^fl/fl^* mice did not induce DI phenotypes, whereas simultaneously performed experiments injecting the retrograde AAVrg-Cre as the positive control exerted effects (Supplemental Figure 7, D–F versus A–C). Of note, the abundance of WNK1 expression in the PVN was approximately 25% relative to that in the OVLT (Supplemental Figure 8).

**Figure 7:**
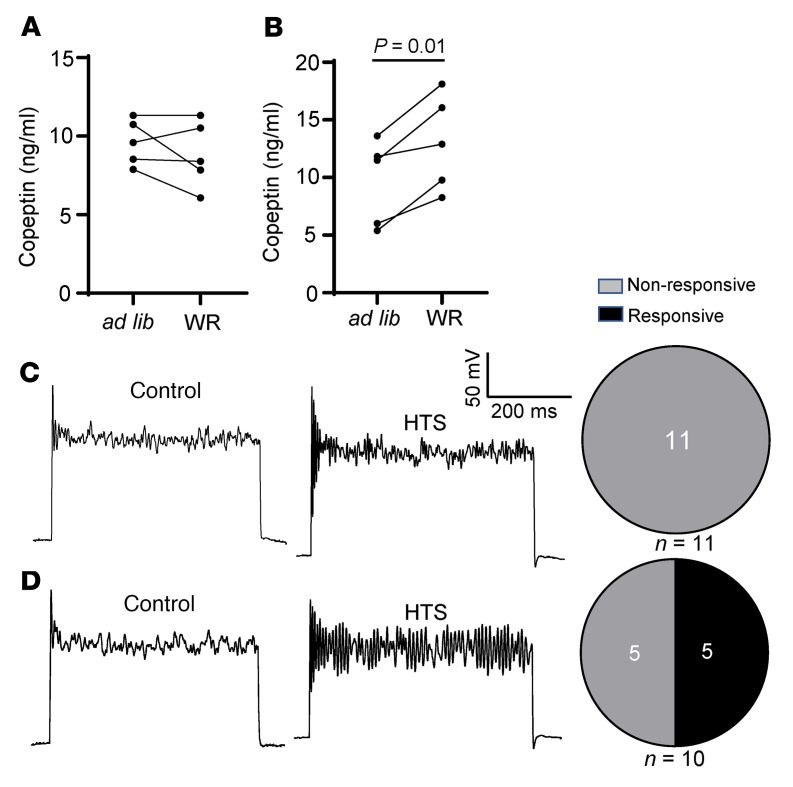
Deletion of *Wnk1* in PVN-projecting OVLT neurons eliminates hypertonicity-induced membrane potential oscillation and blunts copeptin release in response to water restriction. (**A** and **B**) Cooopeptin release in *Wnk1^fl/fl^* and control WT mice with PVN injected with AAV-retro-Cre virus. Statistical comparison was made by paired *t* test between ad libitum and WR. (**C** and **D**) In separate groups of experimental (*Wnk1^fl/fl^*) and control (WT) mice, OVLT neurons were isolated for recording of membrane potential oscillation. Pie charts show distribution of neurons that exhibit membrane potential oscillation responsive and nonresponsive to HTS (5 mM NaCl). *P* < 0.01 between pie chart in **C** and **D** by 2-tailed Fisher’s exact test. In **A** and **B**, *n* = 5 *Wnk1^fl/fl^* and WT mice per experiment, as indicated in scatter plots.

Circulating plasma levels of copeptin were measured in AAVrg-injected mice as well as control mice at day 10 after injection, first at ad libitum and then after 24-hour water restriction (see experimental protocol in Supplemental Figure 6). Deleting WNK1 in CVOs by injecting a retrograde AAVrg-Cre virus in the PVNs of *Wnk1^fl/fl^* mice abolished hypertonicity-induced copeptin release, as evidenced by the lack of differences in the circulating levels between ad libitum and water restriction (Figure 7A). As a positive control, circulating levels of copeptin were increased by water restriction (vs. ad libitum) in the control WT mice with similar retrograde virus injection (Figure 7B). In separate groups of mice, we isolated OVLT neurons for whole-cell current-clamp recording from mice in which *Wnk1* was deleted by injection with a retrograde AAVrg-Cre virus. As is shown in Figure 4E for neurons isolated from *syn1-Cre*–mediated cKO mice, hypertonicity-stimulated membrane potential oscillation was eliminated in mice in which *Wnk1* was deleted in the OVLT by PVN retrograde AAV virus injection (0 of 11 neurons responded, Figure 7C). For control, hypertonicity stimulated membrane potential oscillation in 5 of 10 OVLT neurons isolated from WT mice injected with retrograde AAV virus injection (Figure 7D).

### Activation of Kv3.1 underlies hypertonicity-stimulated AVP release and water homeostasis.

**Figure 8:**
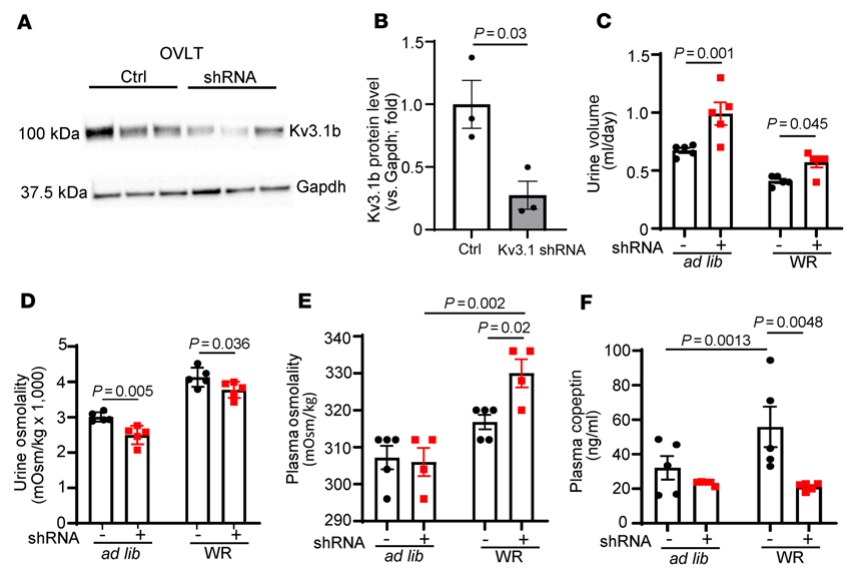
Knockdown of Kv3.1 by shRNA in OVLT causes partial central diabetes insipidus and impairs copeptin release in response to water restriction. (**A**) OVLT tissues from mice with direct injection scrambled RNA (Ctrl) or shRNA against Kv3.1 were probed by antibody against Kv3.1b. Note that the Kv3.1 shRNA targets both alternatively spliced Kv3.1a and Kv3.1b isoforms. (**B**) Mean ± SEM of Kv3.1b protein abundance from 3 separate experiments as shown in **A** (data from each experiment is the average of triplicate samples). Statistical analysis by unpaired *t* test. (**C**) Urine volume, (**D**) urine osmolality, (**E**) plasma osmolality, and (**F**) copeptin levels of mice injected with control scrambled RNA (labeled –) or shRNA against Kv3.1b (labeled +) into OVLT and at either ad libitum water intake or after 24-hour water restriction (WR). Unpaired *t* test for comparison between control scrambled RNA and Kv3.1 shRNA in **B**. In **C**–**F**, *n* = 5 mice per group injected with control scrambled or with Kv3.1b shRNA as indicated in scatter plots. Statistical analysis was performed with by 2-way repeated ANOVA with Šidák post hoc analysis.

The results of high activation threshold and slow inactivation kinetics and inhibition by the extracellular TEA in whole-cell current-clamp recording of freshly isolated neurons suggest that channels of the Kv3 family are involved. Quantitative real-time PCR analysis revealed Kv3.1b was the predominant subtype in OVLT neurons (Supplemental Figure 9). We knocked down Kv3.1b in OVLT neurons by direct injection of AAV virus encoding shRNA against Kv3.1 gene. Successful knockdown of Kv3.1b in OVLT was evident by marked reduction of Kv3.1b protein in the OVLT but not in adjacent brain region (Figure 8, A and B, and Supplemental Figure 10). Compared with mice injected with control scrambled RNA, mice injected with anti-Kv3.1 shRNA developed polyuria and relative hypotonic urine during ad libitum water intake, which persisted during water restriction (Figure 8, C and D). Plasma osmolality was not different between mice with control scrambled RNA and with Kv3.1 shRNA during ad libitum water intake, but plasma osmolality became significantly different after water restriction (Figure 8E). As expected, water restriction increased circulating copeptin levels in control mice (Figure 8F). Knocking down Kv3.1b abolished the WR-induced increase, supporting its role in mediating hypertonicity stimulation of AVP release. For phenotype comparison, we also performed experiments by direct injection of an AAV virus carrying shRNA against *Wnk1* gene. Mice with knockdown of WNK1 in the OVLT exhibited partial DI phenotypes, as in mice with knockdown of Kv3.1b (Supplemental Figure 11), supporting the notion that WNK1 and Kv3.1 act in the same pathway.

### Activation of WNK1 in CVOs stimulates AVP release with reduction in urine output and increases in urine osmolality.

Chloride ion (Cl^–^) binding to the activity center of WNK kinases inhibits catalytic activities, and mutation of Cl^–^-binding amino acids in WNKs activates their kinase activity (32). Mice carrying knockin allele of Cl^–^-insensitive WNK4 have phenotypes that are characteristic WNK4 gain of function (33). We generated conditional knockin (cKI) mice carrying a floxed allele of conditionally activatable Cl^–^-insensitive WNK1 (Supplemental Figure 12). The CKI allele carried a reverse-oriented exon 3 with nucleotides coding for double L369 and L371 to phenylalanine mutation (L369F/L371F) that reorientates and becomes active upon Cre-mediated excision.

**Figure 9:**
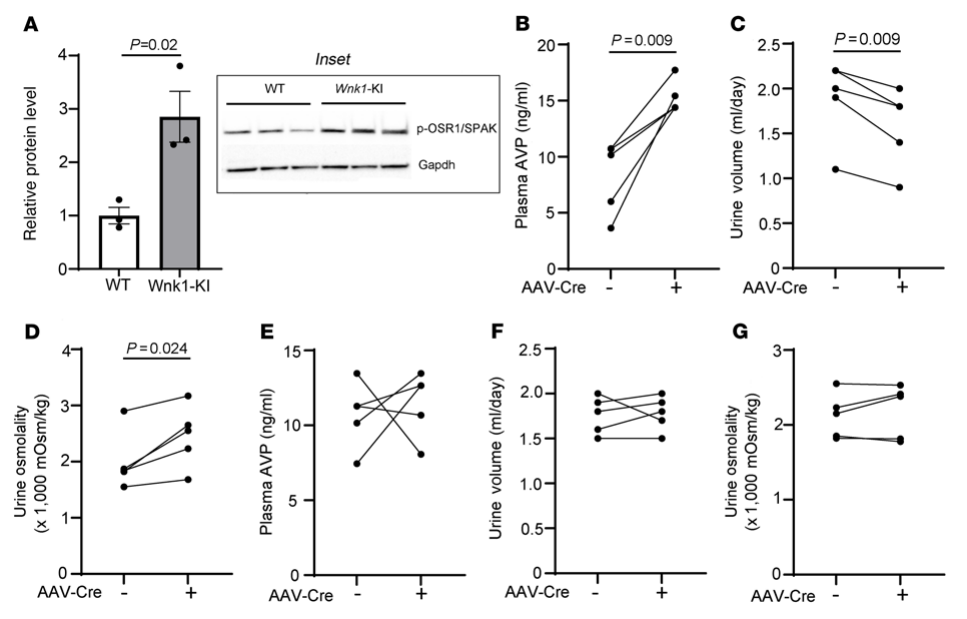
Activation of WNK1 in OVLT increases AVP release. WT mice or mice heterozygous for GOF Cl^–^-insensitive *Wnk1*-knockin (*Wnk1*-KI) allele received AAV-Cre virus injection in OVLT. (**A**) Relative abundance of phospho-OSR/SPAK (p-OSR/SPAK) in KI mice before (–) and after (+) injection, as measured by Western blotting analysis of OVLT using antibody against S373-phospho-SPAK/S325-phospho-OSR1. The inset shows representative Western blotting of 3 separate experiments. Each experiment consists of 3 replicates of WT and 3 *Wnk1*-KI mice. Each data point in the bar graph represents the average of 3 replicates. Statistical analysis by unpaired *t* test. (**B**) Plasma AVP level, (**C**) urine volume, (**D**) urine osmolality in heterozygous *Wnk1*-KI mice in which OVLT was injected with AAV-Cre virus, (**E**) plasma AVP level, (**F**) urine volume, and (**G**) urine osmolality of WT mice in which OVLT was injected with AAV-Cre virus. In **B**–**G**, *n* = 5 mice, as indicated in line plots. Statistical analysis by paired *t* test.

Mice heterozygous for Cl^–^-insensitive *Wnk1*-knockin allele and control WT mice were injected with an AAV-Cre virus in the OVLT. As shown, the activity of WNK1 in OVLT neurons in knockin mice was enhanced, as demonstrated by increased abundance of phospho-OSR1/SPAK (Figure 9A). Plasma AVP levels were increased in cKI mice after AAV-Cre injection compared with those before (Figure 9B). Along with the increases in AVP levels, mice exhibited reduction in urine volume as well as an increase in urine osmolality (Figure 9, C and D). WT mice that received AAV-Cre injection in the OVLT did not exhibit changes in plasma AVP levels or urine volume and urine osmolality (Figure 9, E–G). Thus, an increase in WNK1 kinase activity leads to AVP release.

### Inhibition of WNK1 by pan-WNK inhibitors or genetic deletion of Wnk1 blunts hypertonicity stimulation of AP firing in OVLT neurons.

**Figure 10:**
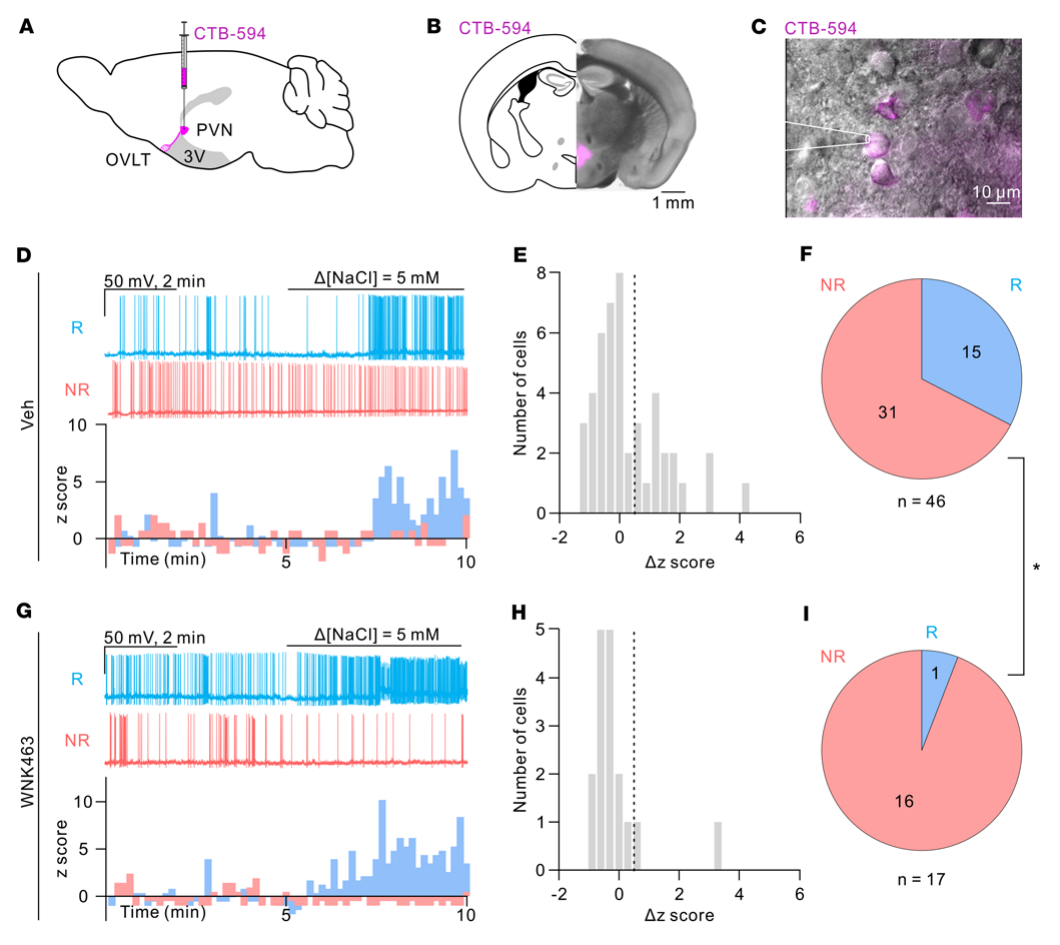
Pharmacological inhibition of WNK1 abolishes hypertonicity-induced spike generation in PVN-projecting OVLT neurons. (**A**) Schematic of the retrograde tracer CTB-594 (Alexa Fluor 594–conjugated recombinant cholera toxin subunit B) injection at the PVN for labeling of PVN-projecting OVLT cells. (**B**) Representative coronal section of the mouse brain injected with CTB-594 at the PVN region. Scale bar: 1 mm. (**C**) Overlay of epifluorescence and IR-DIC images showing CTB-expressing neurons in the OVLT region. Scale bar: 10 μm. A recording pipette attached to a CTB-expressing cell is illustrated. (**D**) Top: Representative traces of the spontaneous firing recorded from a NaCl-responsive (R; cyan trace) neuron and a NaCl-nonresponsive (NR; red trace) neuron. Slices were incubated in the vehicle-containing solution before recording. Bottom: Histogram of *z* score from the representative NaCl-R and NaCl-NR cells. (**E**) Distribution of the *z* score change (Δ*z* score) in response to 5 mM NaCl stimulation of all recorded cells in the vehicle group. The dashed line indicates 0.5. (**F**) Pie chart showing distribution of NaCl-R (Δ*z* score > 0.5) and NaCl-NR (Δ*z* score < 0.5) PVN-projecting OVLT neurons in the vehicle group. (**G**) Top: Representative traces of the spontaneous firing recorded from a NaCl-R neuron and a NaCl-NR neuron. Slices were incubated in the WNK463-containing solution before recording. Bottom: Histogram of *z* score from the representative NaCl-R and NaCl-NR cells. (**H**) Distribution of the Δ*z* score in response to 5 mM NaCl stimulation of all recorded cells in the WNK463 group. The dashed line indicates 0.5. (**I**) Pie chart showing distribution of NaCl-R and NaCl-NR PVN-projecting OVLT neurons in the WNK463 group. *P = 0.048, between **F** and **I**, 2-tailed Fisher’s exact test. The vehicle group consists of recordings of 46 cells from 34 mice. WNK463 consists of 17 cells from 9 mice.

**Figure 11:**
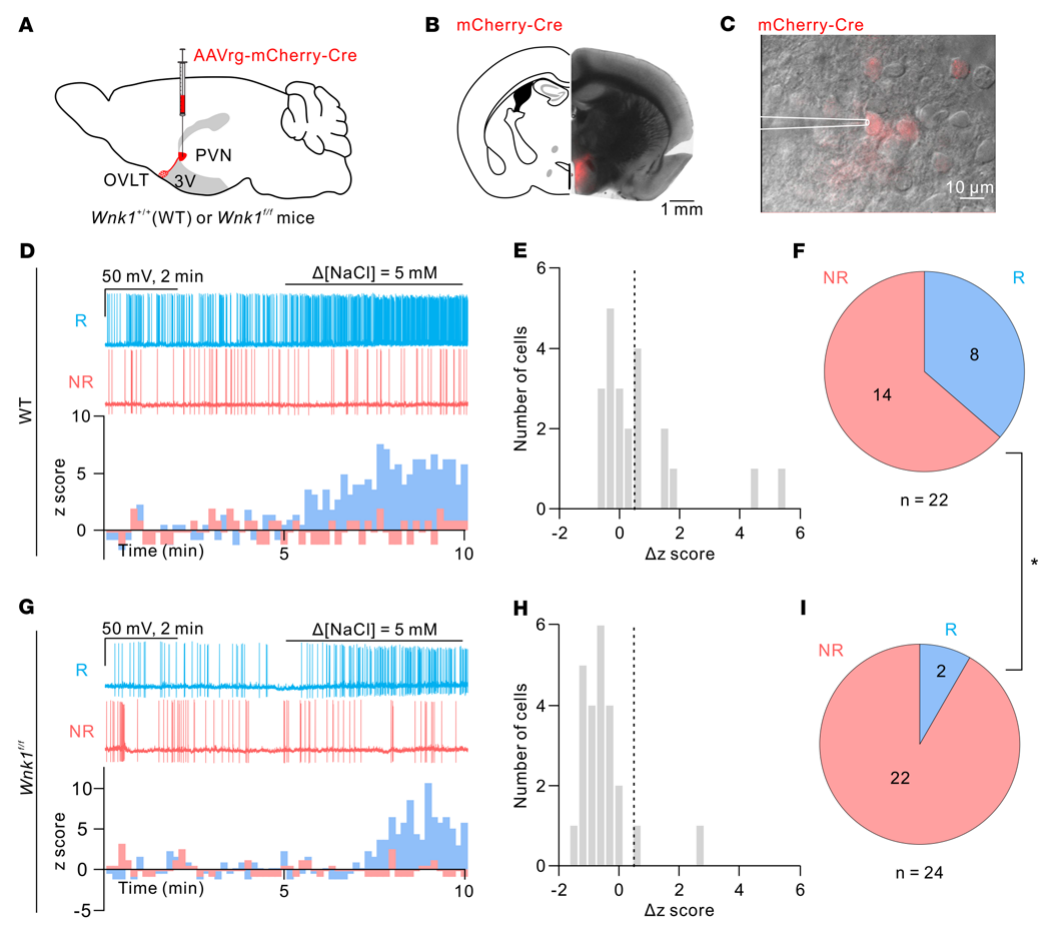
*Wnk1* deletion reduces hypertonicity-induced spike generation in PVN-projecting OVLT neurons. (**A**) Schematic of the virus-mediated KO of *Wnk1* in PVN-projecting OVLT neurons via injection of Cre-expressing retrograde virus at the PVN region. (**B**) Representative coronal section of the mouse brain injected with Cre-expressing virus at the PVN region. Scale bar: 1 mm. (**C**) Overlay of epifluorescence and IR-DIC images showing Cre-expressing neurons in the OVLT region. Scale bar: 10 μm. A recording pipette attached to a Cre-expressing cell was illustrated. (**D**) Top: Representative traces of spontaneous firing recorded from a NaCl-R neuron (R; cyan trace) and a NaCl-NR neuron (NR; red trace) in the WT mice. Bottom: Histogram of *z* score from the representative NaCl-R and NaCl-NR cells. (**E**) Distribution of the Δ*z* score in response to 5 mM NaCl stimulation of all recorded neurons in WT mice. The dashed line indicates 0.5. (**F**) Pie chart showing distribution of NaCl-R and NaCl-NR PVN-projecting OVLT neurons in WT mice. (**G**) Top: Representative traces of spontaneous firing recorded from a NaCl-R neuron and a NaCl-NR neuron in the *Wnk1*–conditional KO (cKO) mice. Bottom: Histogram of *z* score from the representative NaCl-R and NaCl-NR cells. (**H**) Distribution of the Δ*z* score in response to 5 mM NaCl stimulation of all recorded neurons in *Wnk1*-cKO mice. The dashed line indicates 0.5. (**I**) Pie chart showing distribution of NaCl-R and NaCl-NR PVN-projecting OVLT neurons in *Wnk1*-cKO mice. *P = 0.032, between **F** and **I**, 2-tailed Fisher’s exact test. The WT group consists of recordings of 22 cells from 13 mice; the cKO group consists of 24 cells from 11 mice.

Our studies thus far have examined the role of WNK1 in water homeostasis and hypertonicity stimulation of AVP release at the whole-animal level as well as in freshly isolated OVLT neurons. To solidify the conclusion, we further addressed the involvement of the WNK1 using ex vivo recordings of spontaneous APs on the OVLT-containing brain slices. PVN-projecting OVLT neurons were identified with the retrogradely expressed fluorescence signals (Figure 10, A–C). Spontaneous APs could be detected when the OVLT neurons were synaptically isolated with bath application of synaptic blockers (see Methods) and current clamped at approximately –50 ± 5 mV (Figure 10D). Hypertonic challenge (Δ[NaCl] = 5 mM) increased spontaneous AP generation by approximately 33% (15 of 46 cells) in the vehicle-treated neurons (Figure 10, D–F). Preincubation of pan-WNK inhibitor WNK463 (10 μM) for 3 hours significantly reduced the percentage of neurons that responded to hypertonicity (Figure 10, G–I, 1 of 17 cells, 6%; *P* = 0.048, 2-tailed Fisher’s exact test comparing Figure 10, F and I). In addition to pharmacological inhibition of WNK1, we carried out studies in mice in which *Wnk1* in the OVLT was deleted by injecting a retrograde Cre-expressing AAV in the PVN (Figure 11, A–C). Approximately 36% (8 of 22 cells) of control WT neurons increased AP firing in response to hypertonicity stimulation (Figure 11, D–F), while only 8% (2 of 24 cells) of the *Wnk1*-cKO neurons responded to hypertonicity stimulation (Figure 11, G–I; *P* = 0.032, 2-tailed Fisher’s exact test comparing Figure 11, F and I). These data support the notion that WNK1 function is crucial for the osmolality sensing and stimulation of AP generation in PVN-projecting OVLT neurons.

We also confirmed that the stimulatory effect on AP firing is due to hypertonicity, rather than triggered by increases in [Na^+^]. We found that increasing extracellular osmolality via bath application of additional 10 mM mannitol increased AP generation in approximately 45% of OVLT neurons (Supplemental Figure 13). Furthermore, we demonstrated that Kv3 underlies the hypertonicity-induced AP generation: TEA (at 3 mM, which preferentially blocks Kv3) prevented hypertonicity-induced AP generation in OVLT neurons (Supplemental Figure 14). Overall, our results provide compelling support for the notion that activation of Kv3.1 is important for WNK1-mediated regulation of APs in OVLT neurons in response to hypertonicity.

## Discussion

The molecular identity of central osmosensor(s) has not been clearly defined so far. The conventional wisdom favors identifiying resident membrane proteins capable of reading membrane stretch and tension as the osmosensor(s). Earlier studies provided experimental evidence suggesting that mechanosensitive channels TRPV1 and TRPV4 play an important role in the central osmosensing for AVP release and thirst (23, 34–36). Mice with TRPV4 KO exhibited defects in osmolality regulation (37, 38). However, TRPV1 and TRPV4 are activated by stretch. Thus, these channels should be activated by hypotonicity, which causes cell swelling and membrane stretch, rather than by hypertonicity that causes cell shrinkage. To address the conundrum, a later study reported that an alternatively spliced partially N-terminal-deleted TRPV1 isoform (ΔN-TRPV1) acts as a stretch-inhibited channel, and authors proposed that this TRPV1 isoform may potentially be an osmosensor (39). Yet, others show that KO of TRPV1 and/or TRPV4 in mice does not affect AVP release and water drinking (40, 41). A recent study revised the view on the role of TRPV4 and reported that TRPV4 is expressed in glial cells and indirectly mediates hypotonic inhibition of central osmosensation (42).

In the present study, we provide compelling evidence supporting that intracellular protein kinase WNK1 is involved in sensing serum hyperosmolality to regulate AVP release. We showed that mice with neuron-specific *Wnk1* deletion have water homeostasis defects consistent with partial central DI. Using retrograde viral tracing we showed that deletion of *Wnk1* in PVN-projecting CVO neurons is largely responsible for the observed partial central DI phenotypes. Furthermore, OVLT neurons freshly isolated from normal mice responded to hyperosmolality; the response was abolished in *Wnk1*-deleted neurons. Conversely, we showed that activation of WNK1 in OVLT neurons lead to increased circulating AVP levels and changes in urine output and urine osmolality consistent with increased action of AVP on the kidney. The findings that WNK1-mediated osmosensing is reduced by chemical inhibitors of the catalytic activity of WNK kinases as well as studies employing no ATP or nonhydrolyzable ATP analogs in cytosol support that the kinase activity of WNKs is necessary. Whether WNK1 kinase acts directly or through downstream OSR1/SPAK kinase remains to be investigated.

**Figure 12:**
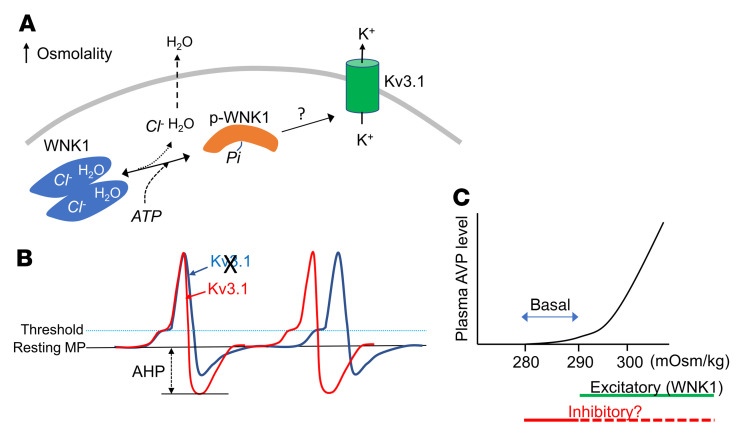
Working model illustrating WNK1 in CVOs as an osmosensor to regulate AVP release via Kv3.1. (**A**) WNK1 exists in conformational equilibrium between chloride-bound autoinhibited dimer and chloride-free activation-competent monomer. Hyperosmolality extracts water from the cell and from the catalytic core of WNK1, which facilitates chloride unbinding, allowing autophosphorylation at S382 and be activated (38–40). WNK1 may activate Kv3.1 directly or indirectly through other intermediaries such as OSR1/SPAK. (**B**) Kv3.1 is a high-threshold voltage-gated K^+^ channel activated by membrane depolarization to –20 mV or above (24, 25). Activation of Kv3.1 shortens action potential duration, increases after hyperpolarization (AHP), and thus increases firing frequency (illustrated by red trace). Conversely, inhibition of Kv3.1 decreases firing frequency (blue trace). In support of this notion, we have found that TEA increased the action potential half-width (data not shown). (**C**) Exponential curvilinear relationship between AVP release and plasma osmolality begins at the threshold of approximately 280 mOsm/kg. WNK1 activation by cellular dehydration (Excitatory pathway; thick green line) plays an important role in AVP release by hyperosmolality. Additional mechanism(s) may be involved, at least for secretion at the basal state, which may include tonic inhibition of osmosensory neurons (Inhibitory pathway; thick solid red line). Loss of hypotonicity-mediated inhibitory pathway (thick dotted red line) may also contribute to hyperosmolality-induced AVP release. Compensation by the additional pathways may account for apparent similar AVP release defects in OVLT-selective deletion of WNK1 (by direct shRNA injection) versus neuronal deletion of WNK1. Extracellular hypertonicity may also activate WNK1 signaling cascade through molecular crowding of the protein (ref. 46) (data not shown).

How does an intracellular protein detect changes in the extracellular tonicity? Structural studies reveal that binding sites for Cl^–^ ions in the catalytic domain and Cl^–^ binding inhibit activation of the kinase (32). Mice carrying amino acid mutation of the Cl^–^ binding site of WNK4 exhibit constitutive activation of the kinase (33), providing direct evidence that WNK kinases are regulated by Cl^–^ at the physiological state. More recent studies further demonstrate that WNKs undergo a solvent-driven (H_2_O-driven) conformational equilibrium between a Cl^–^-bound inactive dimeric state and Cl^–^-unbound activation-competent monomeric state (43, 44). The activity center of WNKs contains many water molecules. Osmotic pressure caused by extracting water from the protein shifted the equilibrium to the latter state (Figure 12A) (43–45). During revision of this paper, a paper reported that hypertonicity induces molecular crowding and phase separation on WNK1, leading to activation of the WNK1-OSR1/SPAK pathway (46). These studies provide a molecular mechanism for how WNKs can function as an osmosensor for the extracellular tonicity.

Osmosensing by CVO neurons relays signal to the PVN and SON through APs. Direct injection of shRNA and neuronal tracing establish that WNK1 in the osmosensory of CVOs is involved. We showed that a physiological range of hyperosmolality (5 mM NaCl) activates K^+^ currents, likely through voltage-gated K^+^ channels, mainly Kv3 family channels. Kv3 channels are high-threshold voltage-activated K^+^ channels that regulate the duration of AP and mediate fast after hyperpolarization (AHP) (29). Activation of Kv3 channels is expected to shorten the AP and increase the AHP, thereby promoting AP firing frequency (Figure 12B). Using single-cell RNA-seq, Pool et al. recently reported that approximately 50% of neurons in CVOs express *Wnk1* or *Kcnc1* gene (coding Kv3.1), and 27%–40% of neurons express both (47). We found that Kv3.1b is the main Kv3 family member in OVLT neurons, and its knockdown leads to impaired osmolality-induced AVP release and water homeostasis defects, as is observed in *Wnk1*-deleted mice. The results support the notion that WNK1 activation of Kv3.1 underlies the electrophysiological basis of CVOs → PVN/SON connection for AVP release.

Neuronal *Wnk1*-deleted mice have blunted AVP release stimulated by water restriction and by mannitol injection. During ad libitum water access, urine output was significantly higher and urine osmolality was lower in *Wnk1*-deleted mice compared with control mice. Basal circulating levels of AVP in *Wnk1*-deleted mice were not detectably different compared with control mice. The relationship of osmolality stimulation to AVP release is exponentially curvilinear (Figure 12C). Sensitivity of detection may contribute to inability to detect differences between *Wnk1*-deleted and control mice at the basal state. Regardless, partial phenotype from *Wnk1* deletion suggests that additional osmosensory molecule(s) or pathways besides WNK1 may exist, at least for basal secretion of AVP. The finding that the effect of pan-WNK inhibitor is not significantly different from that of *Wnk1* deletion suggests that WNK1 is the dominant WNK kinase for central osmosensing for regulating AVP release.

Regarding the potential regulatory pathway or mechanism for basal AVP release, one possibility is that it is through regulation of tonic inhibition by hypotonicity (Figure 12C). Like many regulatory mechanisms in the central nervous system, osmosensation involves dual and reciprocal excitatory and inhibitory pathways. For example, TRPV4 is a stretch-activated channel expresses in glial cells of CVOs that inhibits osmosensation for thirst in response to hypotonicity (42). Hypotonicity increases Ca^2+^ entry through TRPV4 in glial cells, leading to release of taurine, which in turn acts on glycine receptors in osmosensory neurons, causing membrane hyperpolarization and inhibition of AP firing. Whether TRPV4 in glial cells is the mechanism or whether separate mechanism(s) exist in osmosensory neurons to mediate the regulation of basal secretion of AVP requires future investigation.

WNK kinases have been implicated in the cell volume regulation. Cells sense and adapt to extracellular hyperosmolality through RVI to maintain volume constancy. Hyperosmolality activates NKCC1, leading to cellular electrolyte and water entry. This adaptive response results in restoration of cell volume in the setting of continuous existence of extracellular hyperosmolality. Previous in vitro and cell-based studies have shown that WNK1 is activated by hyperosmolality and plays an important role in the RVI following hyperosmolality-induced cell shrinkage. These findings may appear not incongruent with the notion that WNKs are important for central osmosensing. The goal of central osmosensing for the organism is to regulate total body water content to maintain constancy of the extracellular osmolality, rather than to adapt to hyperosmolality. In contrast to peripheral cells, osmosensory neurons in CVOs do not undergo RVI (35, 48). The cell volume of isolated cultured osmosensory neurons decreases in response to a small physiological increase of extracellular tonicity and stays decreased over many hours. This phenomenon prevents signal dampening to ensure proper hydration for the organism. Why osmosensory neurons do not undergo RVI to restore cellular water content is unknown. Nonetheless, the phenomenon allows WNK1 in osmosensory neurons to remain in a water-poor activated state until the extracellular hypertonicity is corrected. It is interesting that the central osmosensory neurons and peripheral cells share a common mechanism of osmosensing.

The intracellular Cl^–^ concentration rises initially during cell shrinkage and subsequently from NKCC1-mediated Cl^–^ entry during RVI (45). How do cells balance the two opposing forces on WNK kinase activity, i.e., inhibition by the high intracellular Cl^–^ concentration and activation by dehydration? Serra et al. recently reported that hyperosmolality activates the LRRC8A-containing Cl^–^ channel (49). It is proposed that Cl^–^ efflux through the channel blunts the rise in cellular Cl^–^ concentration, permitting hyperosmolality-induced cellular dehydration to activate WNK kinase cascade and RVI (45, 49). Lack of RVI in the central osmosensory neurons will diminish NKCC1-mediated Cl^–^ entry. It may be speculated that this fact, plus activation of some Cl^–^ efflux pathway such as LRRC8A, is important for activation of WNK kinases by dehydration.

Na^+^ is the main extracellular cation and determinant of extracellular osmolality. While hypernatremia does activate central osmosensors, a separate sensing system for detecting [Na^+^] is also present in CVOs. The Na^+^ channel Na_x_ (SCN7A) functions as a Na^+^ level sensor by detecting a small (~5 mM) change in [Na^+^] (50). Non-Na^+^ osmolality does not activate Na_x_. By detecting increases in [Na^+^] in blood and CSF, Na_x_ regulates Na^+^ appetite. In our current study, WNK1 osmosensing responds to non-Na^+^ osmolality (mannitol) in isolated neurons as well as in brain slice recording. In vivo data that raising plasma osmolality by mannitol injection (which lowers plasma [Na^+^]) stimulates AVP release in WT mice, but not *Wnk1*-deleted mice, further supports the notion that osmolality is the signal for WNK1 activation, leading to AVP release. Moreover, Na_x_-KO mice have no defects in AVP release (51). Thus, two separate sensing systems for Na^+^ and osmolality allow the body to have independent regulation of body fluid-volume (Na^+^) and water (osmolality), respectively.

Thirst and fluid drinking may be anticipatory or homeostatic. The homeostatic mechanism responds to hypertonicity (osmotic thirst) or hypovolemia (hypovolemic thirst) (6, 21, 27). Whether sensors for osmotic thirst and hyperosmolality-induced AVP release are the same is unknown (21). Previous studies have identified SLC9A4 in OVLT neurons and Na_x_ in glial cells of OVLT as Na^+^ sensors for water intake (52, 53). Additional unknown osmosensors for non-Na^+^ osmolytes in OVLT that mediate water intake have also been proposed (21). In our experiments, SLC9A4 or Na_x_ may conceivably explain compensatory water intake in *Wnk1*-deleted mice during water restriction. The finding that mannitol injection stimulates drinking in *Wnk1*-deleted mice indicates that a non-Na^+^ osmoreceptor for thirst exists and is distinct from WNK1 for AVP release.

In conclusion, we showed that WNK1 plays an important role in detecting and responding to extracellular hypertonicity by releasing AVP. These findings provide fresh insights into how the body senses extracellular tonicity and regulates water homeostasis. Future studies will investigate downstream signaling mechanisms by which WNK1 activates Kv3.1 and the mechanism for regulating basal AVP release in osmosensory neurons.

## Methods

### Animals.

All mice were housed in a temperature-controlled room with a 12-hour-dark/light cycle, with food and water available ad libitum. *Wnk1^fl/fl^* mice have been described previously (16). Synapsin1-cre (strain 003966) and tdTomato-EGFP (mT/mG, strain 007576) mice were from The Jackson Laboratory. Cl^−^-insensitive WNK1 cKI mice were generated by targeted insertion of a cassette containing a reverse-oriented mutant exon 3 (L369F/L371F) and a WT exon 3, flanked by loxP and lox511 sites (see Supplemental Figure 12 for details). Male and female mice with ages between 3 and 5 months were used for the electrophysiological experiments. Age-matched adult male mice were used in metabolic cage experiments.

### Reagents.

The AAVrg-cre retrograde viruses were from Addgene (catalog 24593-AAVrg and 55632-AAVrg) (54, 55). AAV2/5-EGFP and AAV-Cre were from the Viral Vector Core, University of Iowa. Kv3.1 shRNA lentiviral particles (sc-42720-V), WNK1 shRNA lentiviral particles (sc-39257-V), and control shRNA lentiviral particles (sc-108080) were from Santa Cruz. The following primary and secondary antibodies were used: anti-WNK1 (NB600-225 and AF2849, Novus Biologicals); anti-β3 tubulin (MAB1195, R&D Systems); p-WNK1 (pS382) antibody (SPC-1097, StressMarq); anti-Kv3.1 (NBP2-12903, Novus Biologicals), anti–GAPDH-HRP (sc-47724 HRP, Santa Cruz). Alexa Fluor secondary antibodies (A-11005, A-11001, A-11012) were from Thermo Fisher Scientific. Anti-rabbit IgG-HRP (4030-05, Southernbiotech), anti-mouse IgG-HRP (1030-05, Southernbiotech), anti-goat IgG-HRP (6425-05, Southernbiotech), normal Rabbit IgG (catalog 2729, Cell Signaling), normal mouse IgG (sc-2025, Santa Cruz) were used. Angiotensin II (A9525), anti-phospho-SPAK/OSR1 antibody (07-2273), TEA, ATP, AMP-PNP, and ATPγS were obtained from Sigma-Aldrich.

### Immunostaining.

Mice were euthanized and perfused with cold PBS and 4% PFA. The brains were dissected and fixed in 2% PFA overnight, processed through 10% and 30% sucrose, embedded with OCT, and frozen. Frozen sections were cut coronally at 25–30 μm thickness. Brain sections were blocked by 4% normal goat serum and 1% BSA to 0.3% Tris-Triton solution for 2 hours and then incubated with the primary antibody in blocking buffer overnight at 4°C. The sections were washed by PBS containing 0.3% Tris-Triton 3 times and then incubated with second antibodies at room temperature for 1.5 hour. The slides were then washed and mounted and examined with Olympus BX61 microscope.

### Western blotting.

Dissected mouse OVLT tissues were homogenized in RIPA buffer with protease and phosphatase inhibitor cocktail (Sigma-Aldrich) with gentle shaking for 1 hour. The whole lysate was centrifuged at 15,700 g for 30 minutes at 4°C. The supernatant was collected and its protein concentration was determined by BCA. For Western blotting analysis, lysate samples (~20 μg protein) were heated for 5 minutes in 4× SDS-PAGE sample buffer (4% SDS, 20% glycerol, 10% 2-mercaptoethanol, 0.004% bromophenol blue and 0.125 M Tris HCl, pH 6.8) and then separated by precast 4%–12% Bis-Tris Gels (NuPAGE) and transferred to PVDF membrane by electroblotting. The membranes were incubated in blocking buffer (5% milk + Tween-20) for 1 hour followed by incubation with primary antibody diluted in blocking buffer at 4°C overnight. The membranes were washed in TBS containing 0.1% Tween-20 before incubation with a secondary antibody. Bound antibodies were detected using ECL Substrates (Bio-Rad). The bands were quantified by densitometry using ImageJ (NIH).

To detect p-WNK1, WNK1 protein in lysates was immunoprecipitated by anti-WNK1 antibody and probed by antibody against p-WNK1. OVLT tissues from mice were homogenized in nondenaturing lysis buffer (137 mM NaCl, 1% NP-40, 2 mM EDTA, 20 mM Tris HCl, pH 8) with protease and phosphatase inhibitor cocktail (Sigma-Aldrich) and incubated for 2 hours at 4°C before centrifugation for 20 minutes at 13,000*g* and 4°C. The resultant supernatant was incubated overnight at 4°C with 4–5 μL anti-WNK1 antibody at a dilution of 1:200. Then, 40 μL Protein G-Sepharose (Abcam) was added, and incubation was continued at 4°C under rotary agitation for 4 hour. Sepharose was then washed 4 times with lysis buffer (with inhibitors) and centrifuged. The supernatant was removed, and 35 μL PAGE sample buffer was added and incubated at 70°C for 10 minutes before being subjected to Western blotting with the p-WNK1(S382) antibody.

### Metabolic cage studies, urine, and blood analysis.

Mouse metabolic cages (Hatteras Instruments) were used for urine collection and water intake measurement. Urine volume and water intake measurements represent the average of 2–3 experiments. After stereotaxic injection, the mice were allowed to recover for at least 7 days before urine and water intake were measured. The water bottle was removed from metabolic cage for water deprivation. Blood samples were collected by retro-orbital bleeding under anesthesia. Urine and plasma osmolality were measured with a OsmoPRO Multi-Sample Micro-Osmometer (Advanced Osmometer Instruments). Plasma vasopressin and copeptin were measured by ELISA kits (Enzo and Raybiotech, respectively), following the manufacturer’s protocol. For mannitol injection, 0.5 mL of 2 M mannitol or vehicle (water) was injected intraperitoneally into mice (~30 g bodyweight). Water intake and urine volume were measured at 120 minutes. In a separate group of mice, plasma and OVLT tissues were collected at 30 minutes after injection. Plasma Na^+^ concentration was measured using flame photometer.

### RNA isolation and RT‑qPCR.

RNA was extracted from OVLT neurons with the RNAqueous-Micro Total RNA Isolation Kit, and from OVLT tissue by Trizol (Invitrogen). RNA samples were then treated with TURBO DNase (catalog AM1907, Invitrogen) prior to cDNA synthesis using a iScript cDNA Kit (Bio-Rad). cDNA was diluted and real-time qPCR was performed with SYBR mix (Bio-Rad). The following primers were used: Kv3.1a F, TCTCCATTTTGGGAAGCCCC, and R, TCATGCGATAACCCTCAGGC; Kv3.1b F, CGACAGAGGCTGTGAGAGTG, and R, TACTCTGTCCAGGGGTGAGG. The Kv3.2, Kv3.3, Kv3.4, and actin primers are as described previously (56).

### OVLT neuron isolation and electrophysiology.

OVLT neurons were isolated as described previously (22). Briefly, mice were anesthetized and killed by decapitation, and brains were quickly removed and placed in cold (4°C) oxygenated HBSS solution (14175095, Gibco; pH 7.3). The OVLT-containing tissues (~1 mm^3^), located rostral and dorsal to the preoptic recess of the third ventricle, were dissected and incubated in HBSS solution containing 2 mg/mL papain (LS003119, Worthington) at 37°C for 30 minutes, after which they were washed in enzyme-free HBSS solution and filtered by 100 μm filter mesh. The solution was centrifuged at 100*g* for 5 minutes, and the resulting pellet was suspended by 10% FBS+DMEM. The suspension was plated onto laminin- and PDL-coated coverslips for 2 hours, and then cells were used for patch clamping.

Whole-cell recordings were performed as described previously (57). Axopatch 200B patch-clamp amplifier and Pulse software (Molecular Devices) were used to amplify and record currents and potentials. Low-pass currents were filtered at 2 kHz and sampled every 0.1 milliseconds. Data acquisition was performed using a pClamp9.2 program (Axon Instrument Inc.). The pipette resistance was approximately 5–7 MΩ when filled with the pipette solution. Whole-cell access resistance was less than 20 MΩ. Current clamp experiments to record neural oscillations were performed in whole-cell patch mode with extracellular solution containing 140 mM NaCl, 1.8 mM CaCl_2_, 5 mM KCL, 1 mM MgCl_2_, 5.5 mM glucose, 0.33 mM NaH_2_PO_4_, 10 mM HEPES (pH 7.4 with NaOH); intracellular solution contained 130 mM KAc, 1 mM MgCl_2_, 2 mM ATP-Mg, 10 mM EGTA, 0.1 mM GTP, 10 mM HEPES (pH 7.2 with KOH), 4 mM CaCl_2_ (100 nM free CaCl_2_ buffering determined with Maxchelator, Stanford University). Cells were constantly perfused by normal extracellular solution with current injection (600–800 pA) to record the oscillations in the resting potential for 500 milliseconds. To test the effect of hypertonicity on the spontaneous firing in PVN-projecting OVLT neurons, an additional 5 mM NaCl or 10 mM mannitol was added to increase the osmolality in bath solution by approximately 10 mOsm. The spontaneous APs of PVN-projecting OVLT neurons were recorded before and after elevation of extracellular tonicity.

### Stereotaxic injection.

For stereotaxic injections, the mice were anesthetized with either 4% isoflurane inhalation or ketamine/xylazine injection. Scalps were shaved, and the mice were placed in the stereotaxic frame (IVM-3000, Scientifica). For isoflurane anesthesia, the face of each mouse was immersed into the anesthetizing masks supplying with 1.5% isoflurane throughout the surgery. The body temperature of each mouse was maintained at 34°C–36°C using a physiological-biological temperature controller pad (TMP5b, SuperTech Instruments) placed under their body. The head was secured with two ear bars, the surgical area of scalp was sterilized and disinfected with 75% ethanol, and the animal’s eyes were protected by an optical gel. A 10 μL NanoFil syringe (World Precision Instruments) equipped with a 35-gauge beveled metal needle was inserted and positioned. The injections were performed using a nanopump controller (KD Scientific) at a speed of 0.1 μL/min for 2 minutes. After injection, the needle was retained for 5 minutes and then withdrawn slowly. All animals were allowed at least 7 days for complete recovery.

The coordinates for injections were as follows: bilateral PVN (AP, –0.8 mm; ML, ±0.25 mm; DV, 5.3 mm; relative to the bregma, modified from Nomura et al., ref. 58); OVLT (AP, 0.76 mm; ML, ± 0 mm; DV, 4.15 mm, relative to bregma, ref. 59). The retrograde tracer Alexa Fluor 594–conjugated recombinant cholera toxin subunit B (CTB594; catalog C22842, Thermo Fisher Scientific) was injected at 0.2 μL, 1% wt/v. The retrograde AAV-cre, AAV-cre, and control AAV-GFP viruses were injected at 0.2 μL, approximately 1 × 10¹³ virus genome/mL. Virus carrying control or shRNA to Kv3.1 or WNK1 were injected at 0.32 μL, 1 × 10^6^ virus genome/mL.

### Ex vivo patch-clamp recording in OVLT slices.

Acute brain slices were prepared at least 1 week after the retrograde tracer injection or 3 weeks after the virus injection, and recording was performed as previously described (60). All animals were sacrificed by decapitation after anesthesia with isoflurane. Mouse brains were rapidly removed, and coronal brain sections of 300 μm thickness containing the OVLT were sliced in the ice-cold oxygenated (95% O_2_ and 5% CO_2_) sucrose-based solution containing the following: 87 mM NaCl, 75 mM sucrose, 25 mM NaHCO_3_, 10 mM glucose, 7 mM MgCl_2_, 2.5 mM KCl, 1.25 mM NaH_2_PO_4_, and 0.5 mM CaCl_2_ using a microslicer (DTK-1000, Dosaka). For virus-injected mice, brain slices were immediately incubated in the holding chamber containing the same solution at 34°C for 30 minutes after sectioning and kept in the same chamber at the room temperature (23°C ± 2°C) until use. For CTB-injected mice, brain slices were immediately incubated in the holding chamber containing the sucrose-based solution with 10 μM WNK463 or 0.1%(v/v) DMSO at 34°C for 30 minutes after sectioning and kept in the same chamber at the room temperature for 150 minutes further incubation before use.

During experiments, slices were placed in a recording chamber and superfused with oxygenated artificial cerebrospinal fluid containing 125 mM NaCl, 25 mM NaHCO_3_, 25 mM glucose, 2.5 mM KCl, 2 mM CaCl_2_, 1.25 mM NaH_2_PO_4_, 1 mM MgCl_2_, and the following synaptic blockers: 2 mM kynurenic acid (catalog K3375, Merck KGaA), 1 μM SR95531 (catalog ab120042, Abcam), and 1 μM CGP55845 (catalog 1248, Tocris Bioscience) at 23°C ± 2°C using a temperature controller (TMP5b, Supertech Instruments). Tracer- or virus-expressing OVLT neurons were confirmed by red fluorescence and visually selected under an infrared and differential interference contrast (IR-DIC) microscope (BX51WI, Olympus) equipped with an infrared-sensitive charge-coupled device camera (C7500-50, Hamamatsu). Whole-cell recordings were performed with a digitizer-equipped (Digidata 1440A, Molecular Devices) amplifier (Axopatch 200B or 700B, Molecular Devices). Recording electrodes with pipette resistance of 4–6 MΩ were prepared from borosilicate glasses with a filament (OD, 1.5 mm; ID, 0.86 mm; GC150F-7.5, Harvard Apparatus) using a vertical puller (PC-10, Narishige) and a microforge (MF-830, Narishige). Recording electrodes were filled with the low Cl^–^ internal solution containing the following: 136.8 mM K-gluconate, 10 mM HEPES, 7.2 mM KCl, 7 mM Na_2_-phosphocreatine, 4 mM MgATP, 0.5 mM Na_3_GTP, 0.2 mM EGTA, and 0.4% (wt/v) biocytin (pH 7.3 adjusted with KOH). For all the recordings, the pipette capacitance was fully compensated and series resistance was compensated to approximately 80% (bandwidth, 1–2 kHz) in the current-clamp configuration. Signals were low-pass filtered at 2 kHz using a 4-pole Bessel filter and sampled at 10 kHz.

### Statistics.

Data are presented as mean ± SEM. Experimental *n* number is illustrated by scatter plot. Statistical comparisons between two groups of data were made using 2-tailed unpaired Student’s *t* test or paired *t* test as specified. Multiple comparisons were determined using 2-way ANOVA followed by Šidák’s or Tukey’s multiple-comparison tests. Electrophysiological data were analyzed using Clampfit 10.7 (Molecular Devices). Numbers of action potential spikes (no. AP) were analyzed using 10-second bins, and *z* scores were calculated by normalizing to the SD of no. AP during 5-minute baseline before elevating extracellular tonicity [*z* = (no. AP – mean no. AP_baseline_)/SD no. AP_baseline_]. The cells with an average *z* score after hypertonicity stimulation (Δ*z* score) of larger than 0.5 were classified as the stimulation-responsive (R) cells. Statistical significance between the responsiveness of each group was tested using 2-tailed Fisher’s exact test. *P* values of less than 0.05 were considered significant.

### Study approval.

All experimental procedures conform to the *Guide for the Care and Use of Laboratory Animals* (National Academies Press, 2011) and were approved by the Institutional Animal Care and Use Committees at the University of Iowa Carver College of Medicine and National Yang Ming Chiao Tung University.

## Author contributions

XJ, JX, CWY, JCC, and CJC designed the study, conducted the experiments, analyzed the data, and participated in writing the paper. CCL and CLH supervised the project and wrote the final paper. All authors approved the final version of the submitted manuscript.

## Supplementary Material

Supplemental data

## Version 1 04/18/2023

In-Press Preview

## Version 2 06/01/2023

Electronic publication
