# Supplementary material for: WNK1 promotes water homeostasis by acting as a central osmolality sensor for arginine vasopressin release
Source: J Clin Invest. 2023 Jun 1;133(11):e164222. doi: 10.1172/JCI164222 (PMC10231991; doi:10.1172/JCI164222)
Supplement: Supplemental data [file jci-133-164222-s074.pdf]

Supplementary Figures

for

WNK1 promotes water homeostasis by acting as a central osmolality sensor for arginine vasopressin release

Xin Jin,[1] Jian Xie,[1] Chia-Wei Yeh,[2] Jen-Chi Chen,[1] Chih-Jen Cheng,[1] Cheng-Chang Lien,[2,3,*] and Chou-Long Huang[1,*]

[1]Department of Medicine, Division of Nephrology, University of Iowa Carver College of Medicine, Iowa City, Iowa, USA; [2]Institute of Neuroscience and [3]Brain Research Center, National Yang Ming Chiao Tung University, Taipei, Taiwan

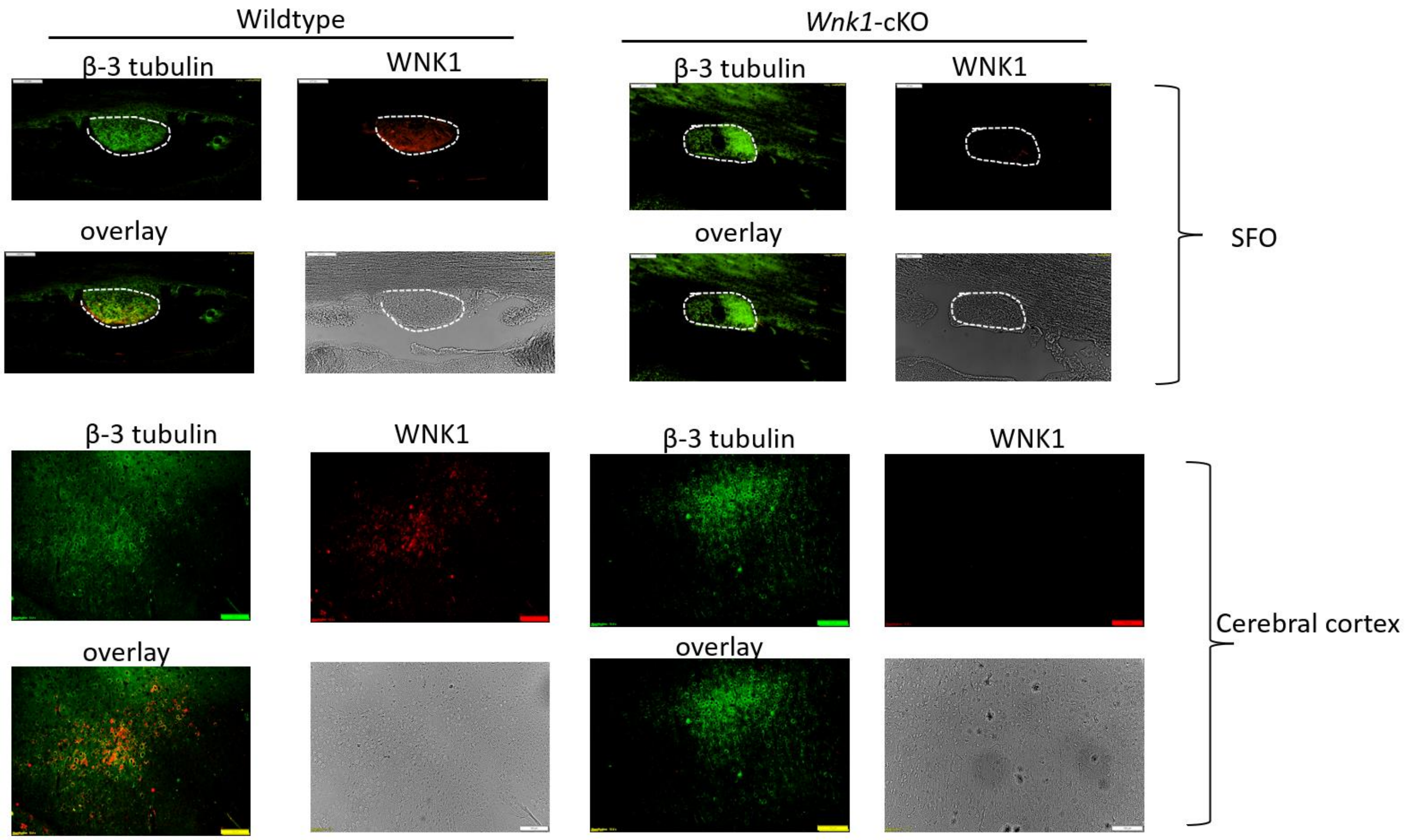

Supplementary Figure 1. ***Wnk1*-cKO mice have reduced WNK1 expression in the cerebral cortex and subfornical organ.** Immunofluorescent staining of WNK1 in subfornical organ (SFO) and cerebral cortex in colocalization with neuronal marker β-3 tubulin in wildtype (WT) and syn1-Cre-mediated *Wnk1*-cKO mice.

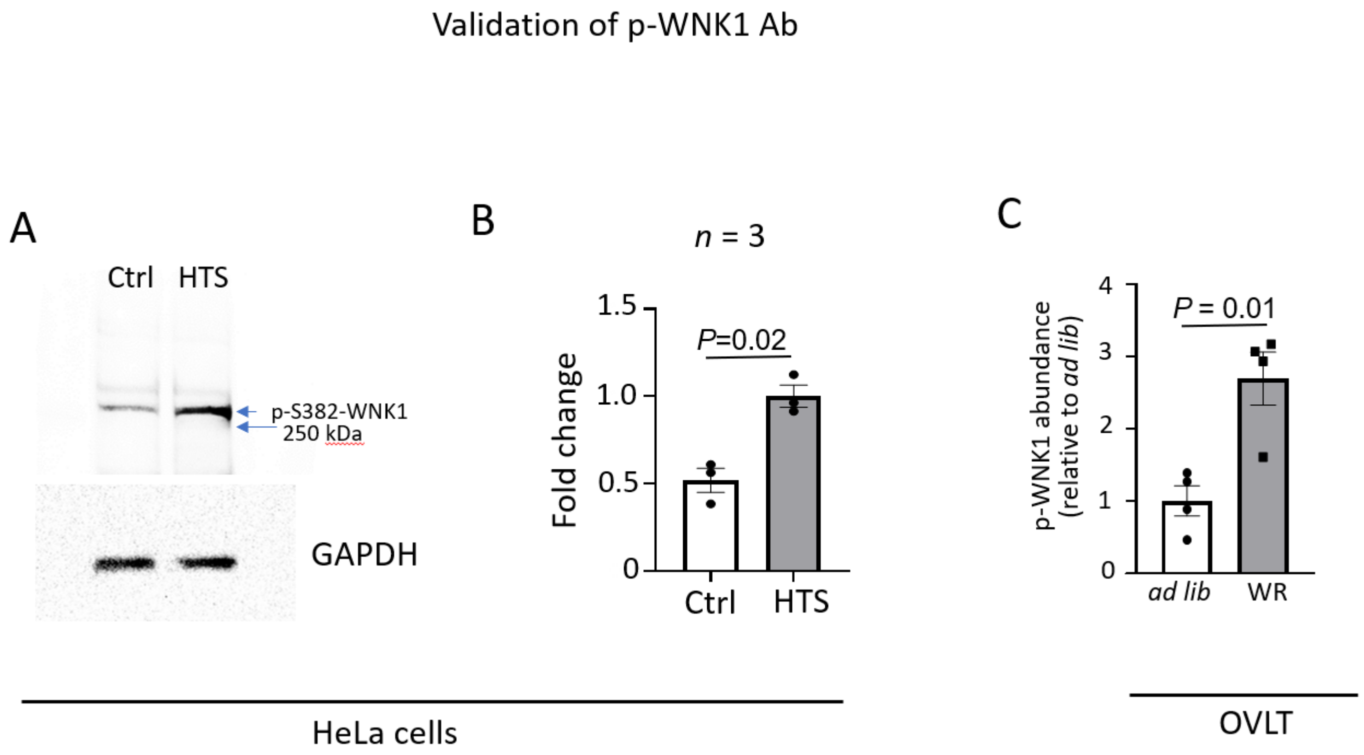

Supplementary Figure 2. Validation of p-WNK1 antibody and increased expression of endogenous p-WNK1 in HeLa cells and in OVLT in response to hypertonicity. (**A**) HeLa cells exposed to culture media with or without 100 mM mannitol (HTS). Western blot analysis probed by antibody against serine-382 phsopho-WNK1 (p-WNK1). Shown is representative of 3 similar experiments. (**B**) Mean ± SEM of results of 3 separate experiment as in panel **A**. Statistical comparison by two-tailed unpaired *t*-test. (**C**) Mean ± SEM p-WNK1 abundance (analyzed by densitometry) of 4 separate experiments as a representative experiment shown in main Figure 2A, *inset*. OVLT tissue lysates from WT mice at *ad lib* water intake and after 24 hr water restriction (WR) were immunoprecipitated by anti-WNK1 antibody and probed by anti-p-WNK1 antibody. *P*=0.01, WR vs *ad lib*. Statistical comparison by two-tailed unpaired *t*-test.

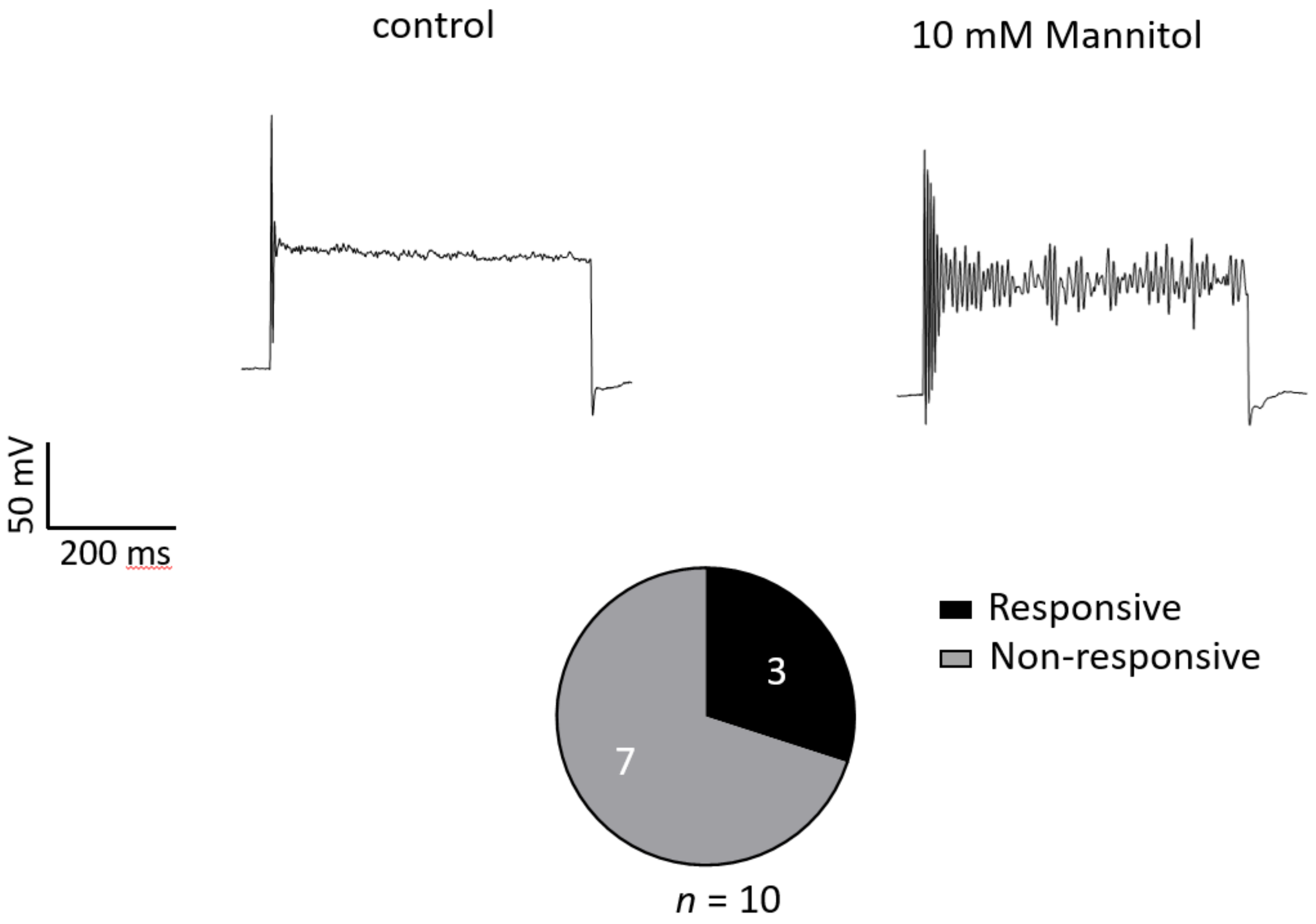

Supplementary Figure 3. Mannitol induces membrane potential oscillation in freshly isolated OVLT neurons. Ruptured whole-cell current-clamp recording for membrane potentials in freshly isolated OVLT neurons at baseline (control) and after incubating with 10 mM mannitol for 3 minutes. Pie chart shows 3 out of 10 recorded neurons responded. For comparison, ~50% neurons responded to 5 mM NaCl and 0% neurons showed oscillation without stimulation by HTS (not shown here).

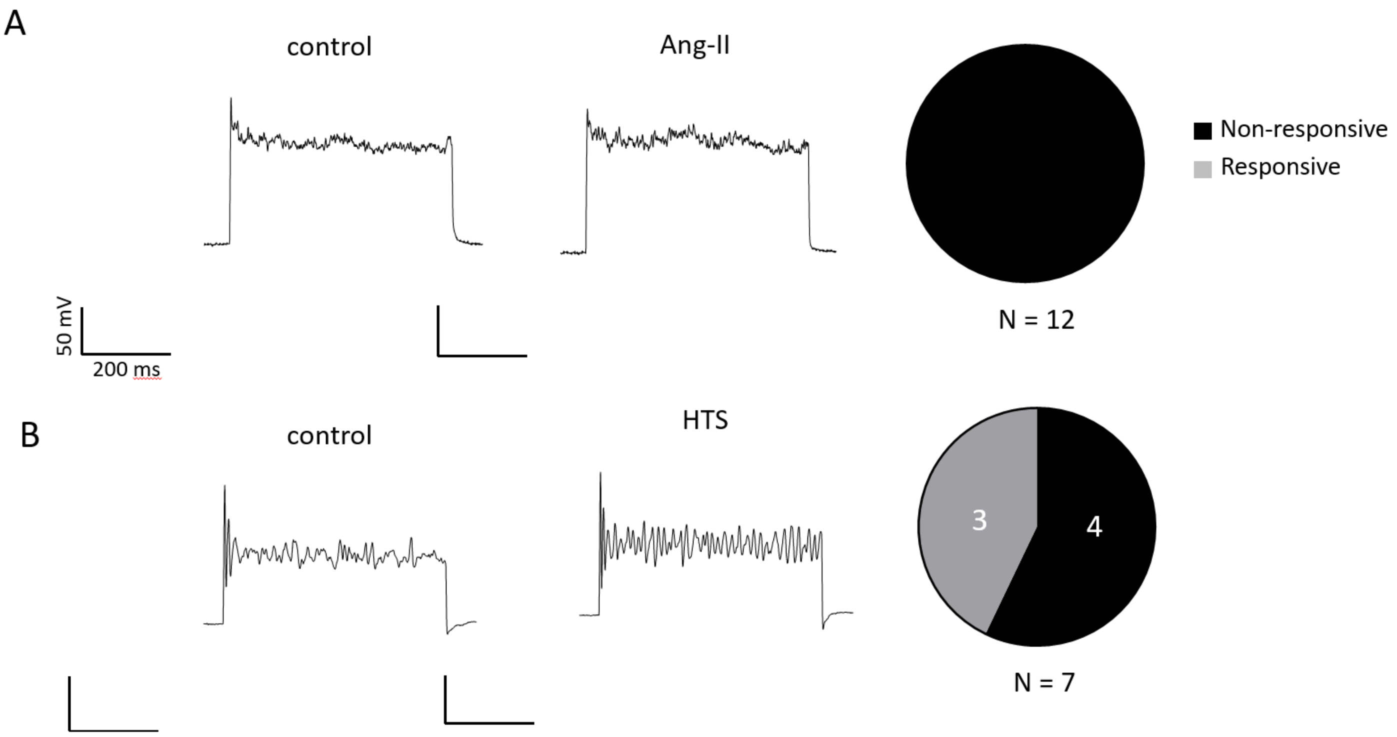

Supplementary Figure 4. Ang II does not induce membrane potential oscillation in freshly isolated OVLT neurons. Membrane potentials of freshly isolated OVLT neurons at baseline, after incubating with 10^{-6} M Ang II (**A**) or 5 mM NaCl (**B**) for 3 minutes, 600 pA currents were injected to depolarize membrane potential from the resting potential -55 mV to +150 mV. Panel **A** and **B** are example of Ang II and NaCl treated neuron, respectively. Pie charts show distribution of responsive and non-responsive neurons. Statistical analysis by two-tailed Fisher’s exact test.

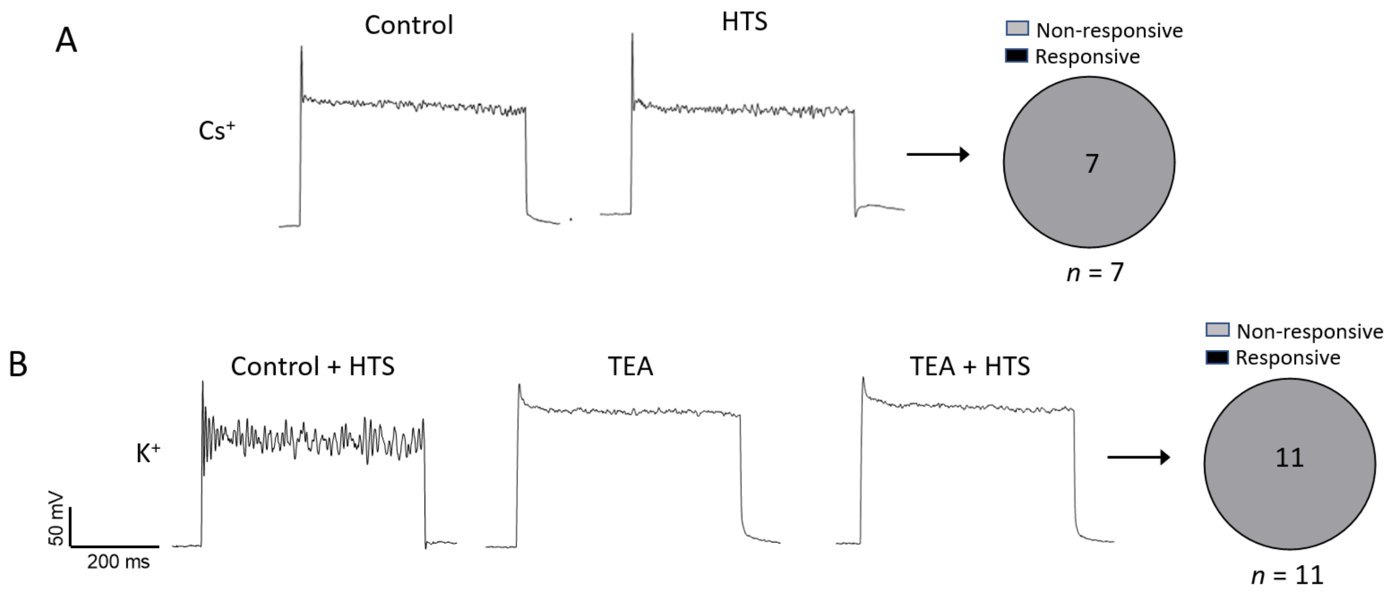

Supplementary Figure 5. Effects of extracellular TEA and substitution of extracellular and intracellular K^+ by Cs^+ on hypertonicity-induced membrane potential oscillation. (**A**) Substitution of K^+ in the bath and pipette by Cs^+ eliminated HTS-induced membrane potential oscillation. Pie chart shows 7 out of 7 neurons with Cs^+ substitution are non-responsive. (**B**) OVLT neurons were bathed with extracellular application of TEA (3 mM) or without (control) before 5 mM NaCl (HTS). Pie chart shows TEA prevented hypertonicity stimulation of membrane potential oscillation in every neuron recorded (11 out of 11 neurons).

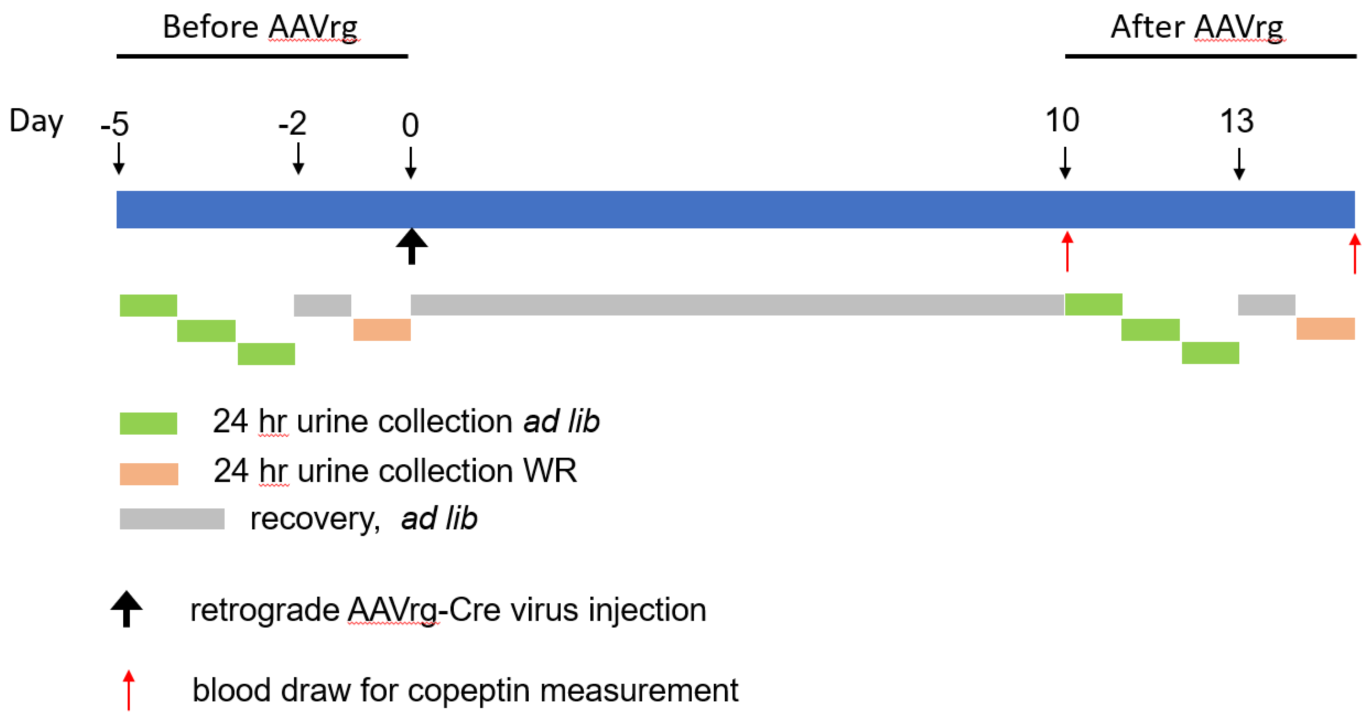

Supplementary Figure 6. Timeline for experiment with AAV-retro-Cre virus injection. Retrograde AAVrg-Cre virus was injected into PVN at day 0. Prior to that urine collection was carried out at *ad lib* for 3 days, followed by 24 hr interval, and under water restriction (WR) for 1 day. Urine output was monitored for ensuing 10 days after injection which exhibited gradual increases (not shown). Thereafter, urine collection was carried out at *ad lib* for 3 days, 24-interval, under water restriction (WR) for a day. A 24-hr day starts from 12 noon to 12 noon next day. Blood was collected via retro-orbital route at day 10 (for *ad lib*) and via intracardiac puncture at end of experiment after euthanasia (for WR).

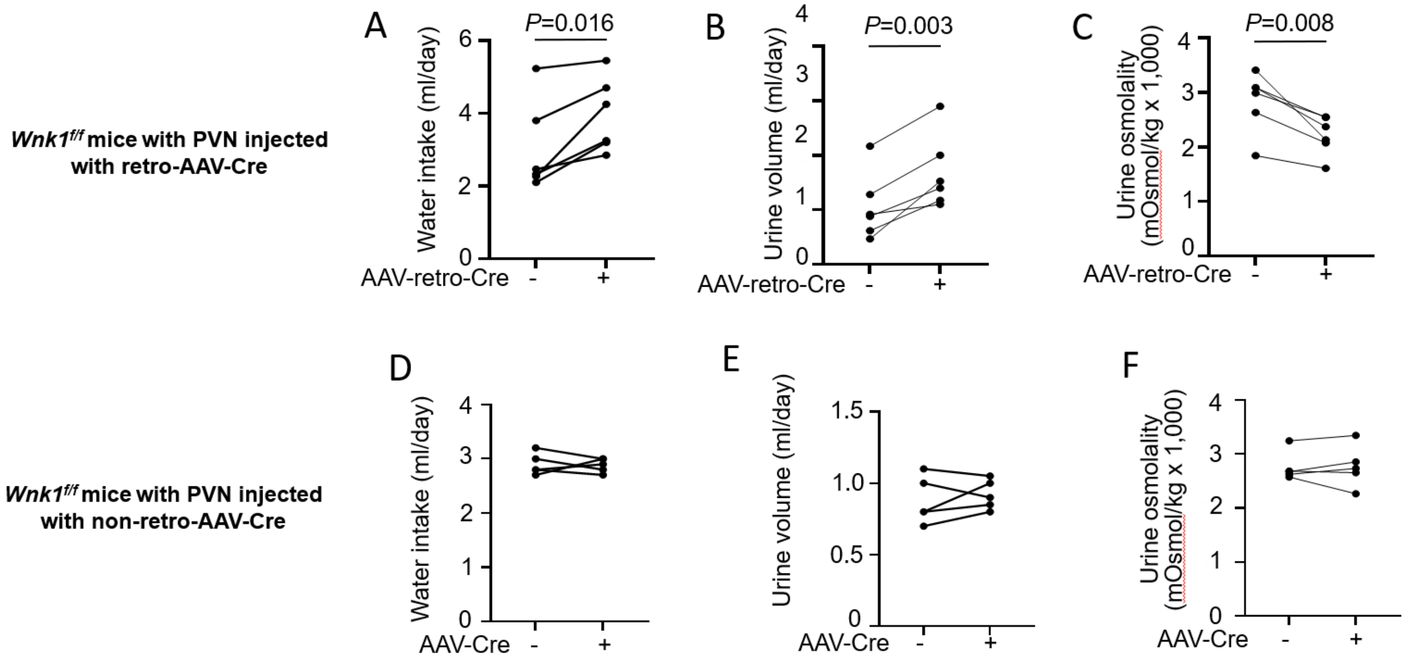

Supplementary Figure 7. Effects of PVN injection of AAV-retro-Cre virus (A-C) vs control non-retro AAV-Cre virus (D-F) into *Wnk1$^{f/f}$* mice. (**A**-**C**) Water intake (**A**), urine volume (**B**), urine osmolality (**C**) of *Wnk1$^{f/f}$* mice before (labeled “-“) and after injection (“+”) with AAV-retro-Cre virus. (**D**-**F**) Water intake (**D**), urine volume (**E**), urine osmolality (**F**) of *Wnk1$^{f/f}$* mice before and after injection with control non-retro AAV-Cre virus into *Wnk1$^{f/f}$* mice. Paired *t*-test for between before and after virus injection. Experimental protocol and timeline as in Supplementary Figure 6.

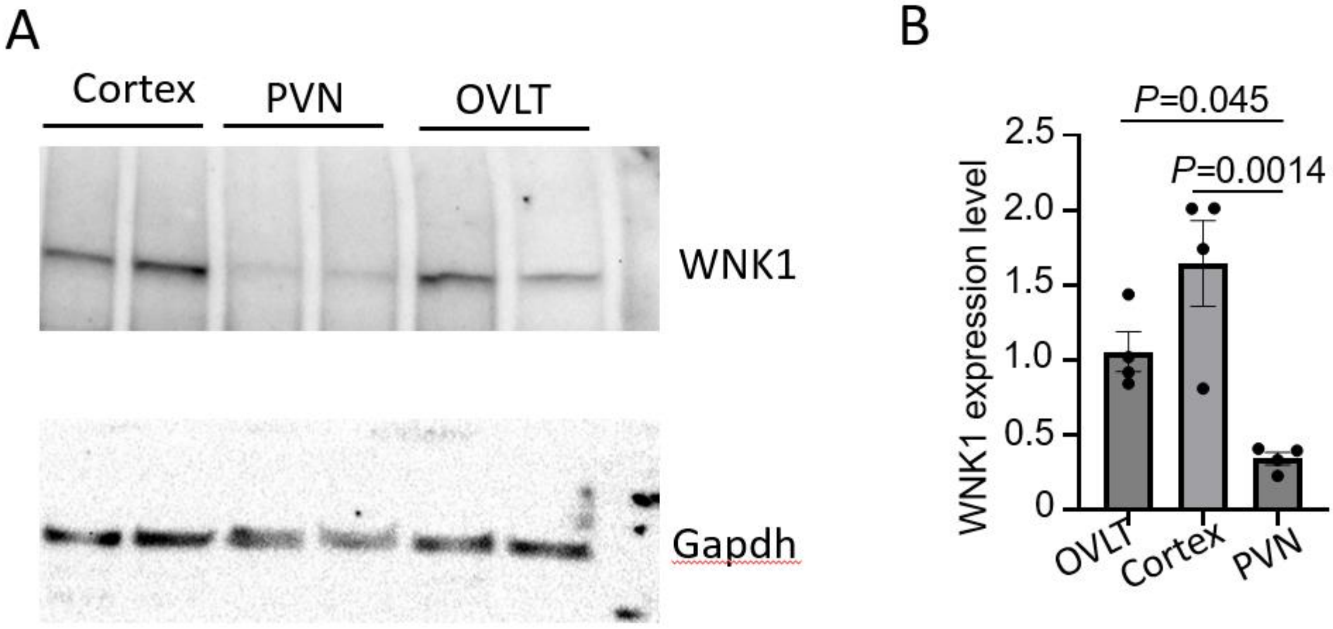

Supplementary Figure 8. Low expression of WNK1 in PVN vs OVLT or cortex. (A) Representative western blot of WNK1 protein in WT of brain regions. (**B**) WNK1 was normalized to Gapdh and compared to OVLT (set as “1”). Mean ± SEM of 3 separate experiments as shown in panel A. Statistical analysis by one-way ANOVA with Tukey post-hoc analysis.

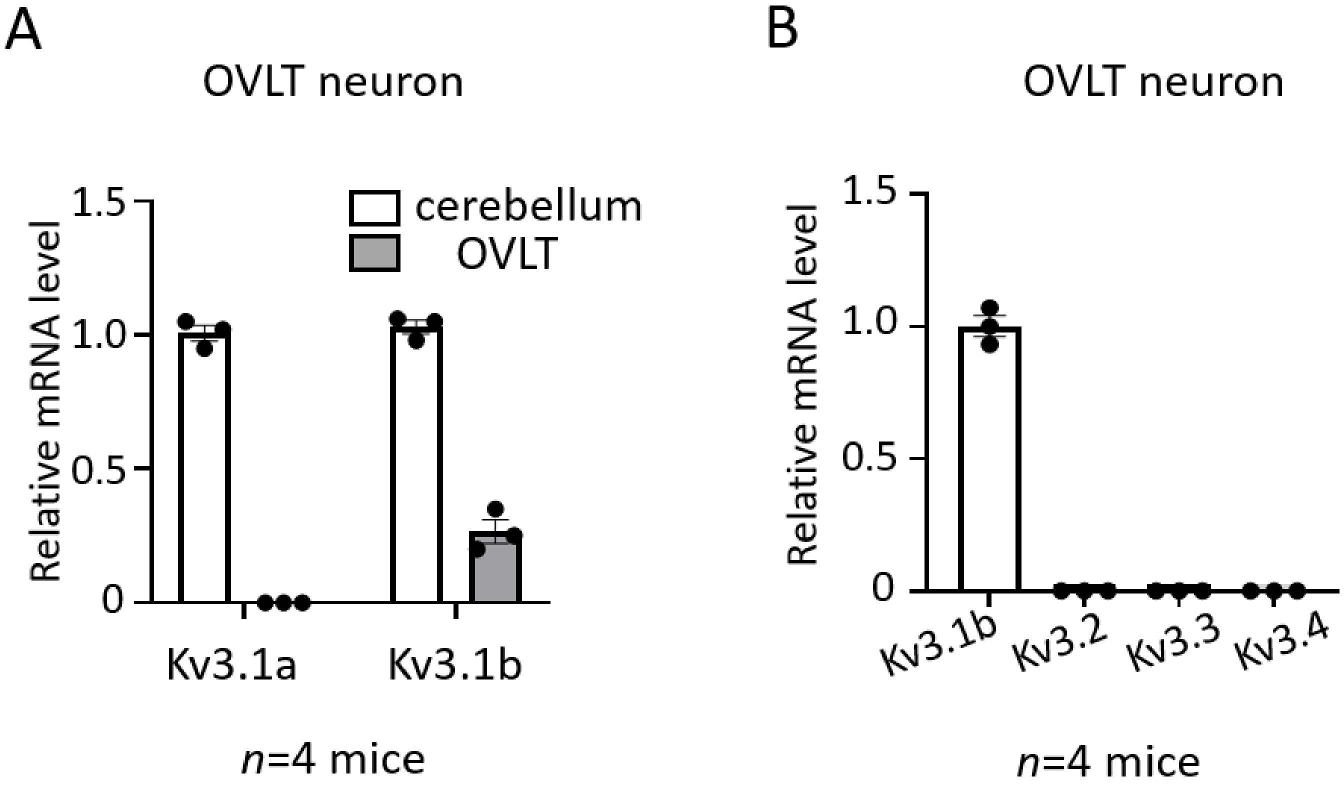

Supplementary Figure 9. Expression of Kv3.1b, but not other Kv3 channels, in OVLT. (**A**) mRNA expression (analyzed by qRT-PCR) of Kv3.1a and Kv3.1b in neurons isolated from cerebellum and OVLT. Expression is normalized to Gapdh and the ratio compared relatively to cerebellum (taken as "1") for each transcript. (**B**) Relative mRNA expression of Kv3.1b, Kv2, Kv3, Kv4 in isolated OVLT neurons. Expression is normalized to Gapdh and the ratio compared relatively to Kv3.1b (taken as "1"). Representative of 4 separate experiments, each with 3 samples.

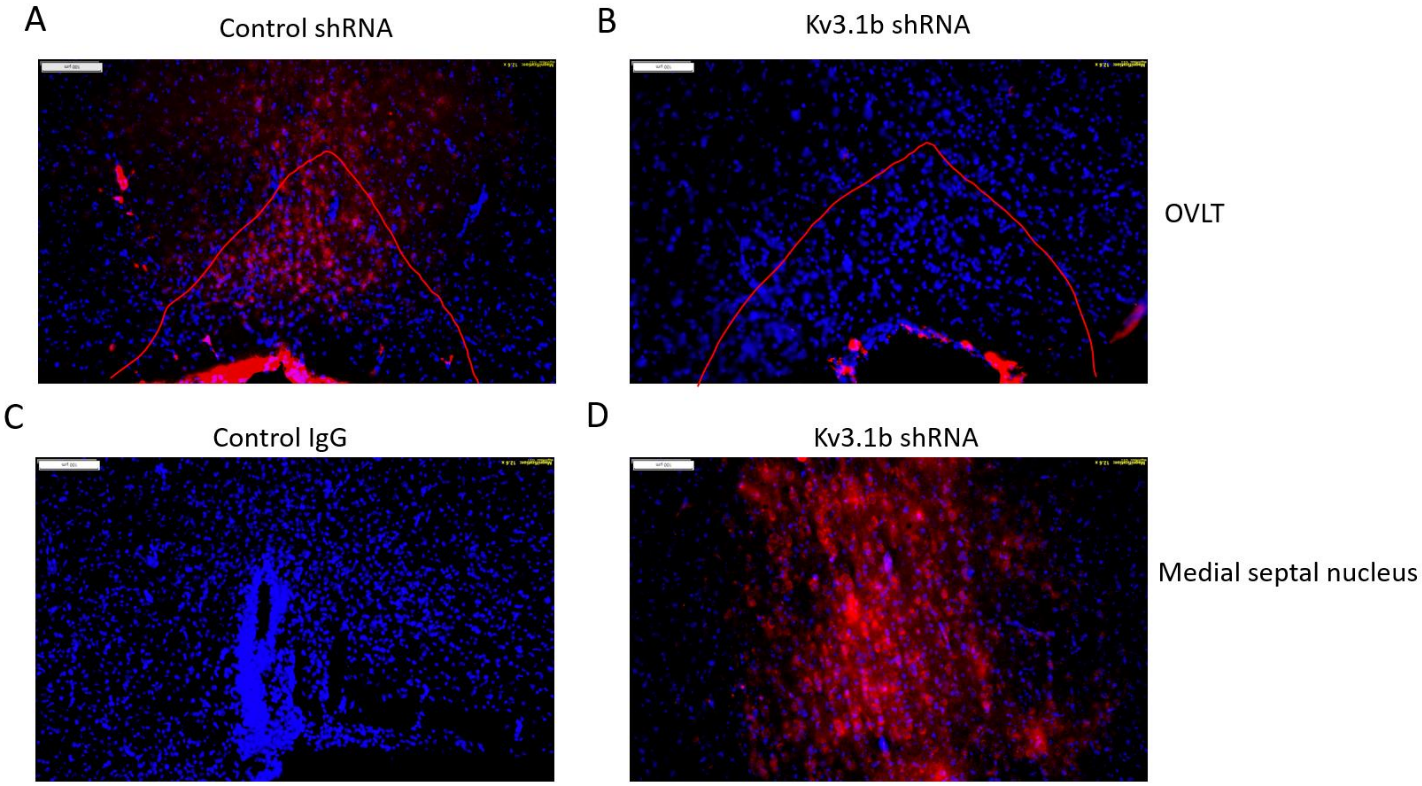

Supplementary Figure 10. immunofluorescent staining of Kv3.1b in OVLT from mice injected with scrambled (control) vs Kv3.1 shRNA. (**A**) Kv3.1b staining in OVLT isolated from mice injected with control scrambled RNA into OVLT. (**B** and **D**) Kv3.1b staining in OVLT and medial septal nucleus isolated from mice injected with Kv3.1 shRNA into OVLT. Note that normal Kv3.1b staining is observed in medial septal nucleus (**D**) while the staining is markedly reduced in OVLT (**B**). (**C**) Medial septal nucleus stained by control IgG serving as a negative control for anti-Kv3.1b antibody.

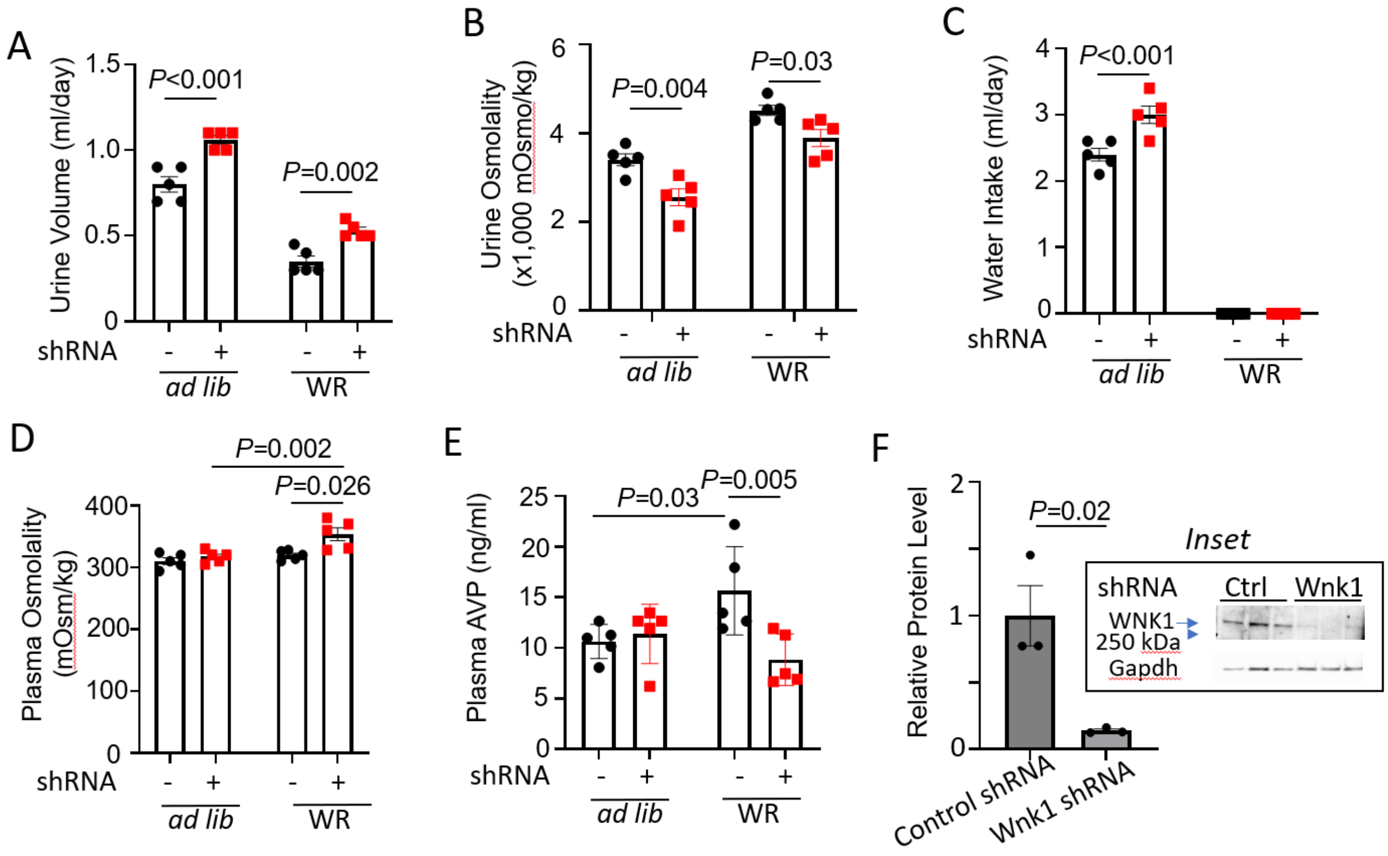

Supplementary Figure 11. Knockdown of WNK1 by shRNA in OVLT causes partial central diabetes insipidus and impairs AVP release in response to water restriction. (**A**-**E**) urine volume (**A**), urine osmolality (**B**), water intake (**C**), plasma osmolality (**D**), and plasma AVP level (**E**), from WT mice injected with control scrambled RNA (labeled "-") or shRNA against WNK1 (labeled "+") into OVLT and at either ad lib water intake or after 24 hr water restriction (WR). statistical analysis by two-way repeated measures ANOVA with Šídák post hoc analysis. (**F**) OVLT tissues from mice with direct injection scrambled RNA (Ctrl) or shRNA against WNK1 were probed by antibody against WNK1 (inset). Mean ± SEM of WNK1 protein abundance from 3 separate experiments as shown in inset (data from each experiment is average of triplicate samples). Statistical analysis by two-way repeated ANOVA with Šídák post-hoc analysis.

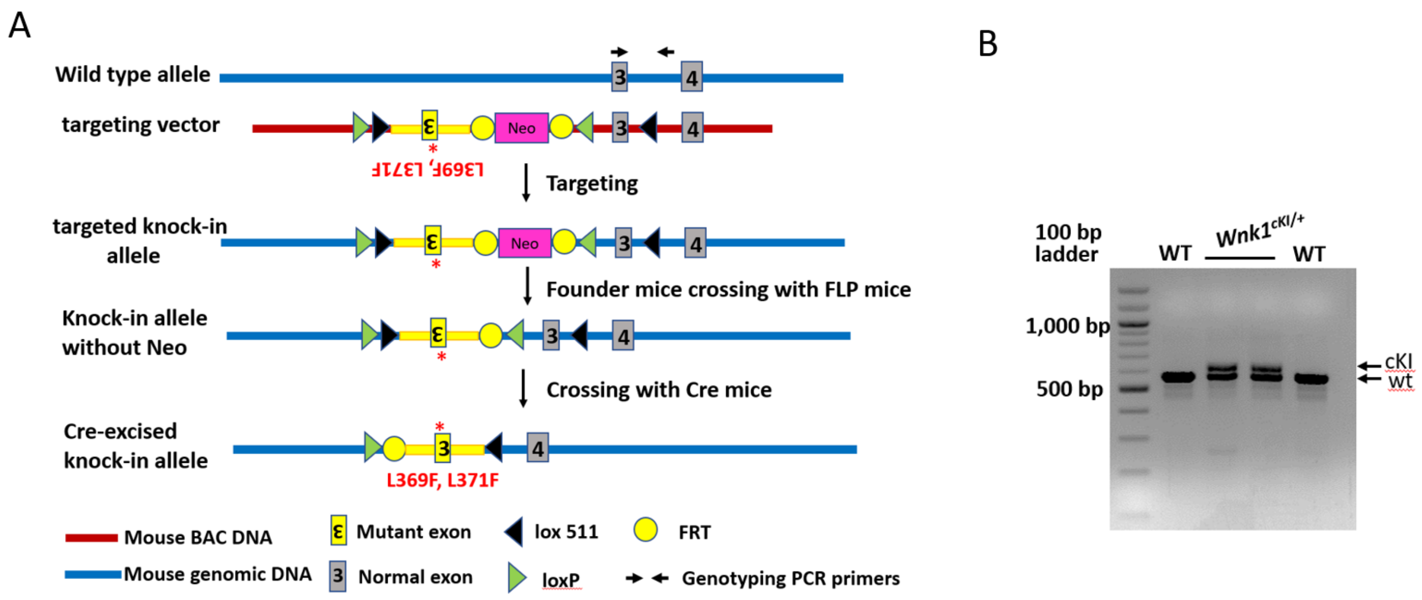

Supplementary Figure 12. Construction of chloride-insensitive *Wnk1* allele and genotyping. (A) Cl^--insensitive *Wnk1* conditional knockin mice were generated by targeted insertion of a cassette containing a reverse-oriented mutant exon 3 (L369F/L371F) and a WT exon 3, flanked by loxP and lox511 sites. Cre excision of knockin allele leads excision of WT exon 3 and re-orientation of mutant exon 3. (**B**) Genotyping using primer sets (as indicated) reveals mice heterozygous for cKI allele have cKI as well WT allele.

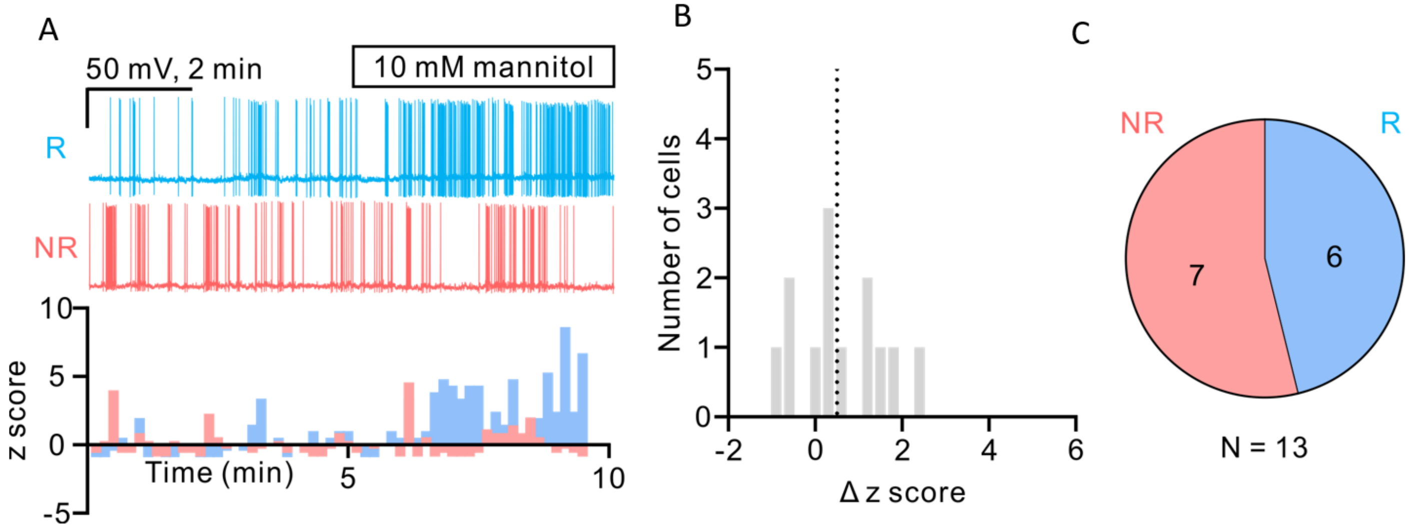

Supplementary Figure 13. Mannitol induces spike generation in PVN-projecting OVLT neurons. (**A**) Top, representative traces of spontaneous firing recorded from a mannitol-R (responsive, cyan trace) neuron and a mannitol-NR (non-responsive, red trace) PVN-projecting OVLT neuron. Bottom, histogram of z score from one each representative mannitol-R and mannitol-NR cell. (**B**) Distribution of the Δz score in response to 10 mM mannitol stimulation of all recorded neurons. Dashed line indicates 0.5. (**C**) Pie chart showing distribution of mannitol-R and mannitol-NR PVN-projecting OVLT neurons. 6 out of 13 neurons respond to mannitol.

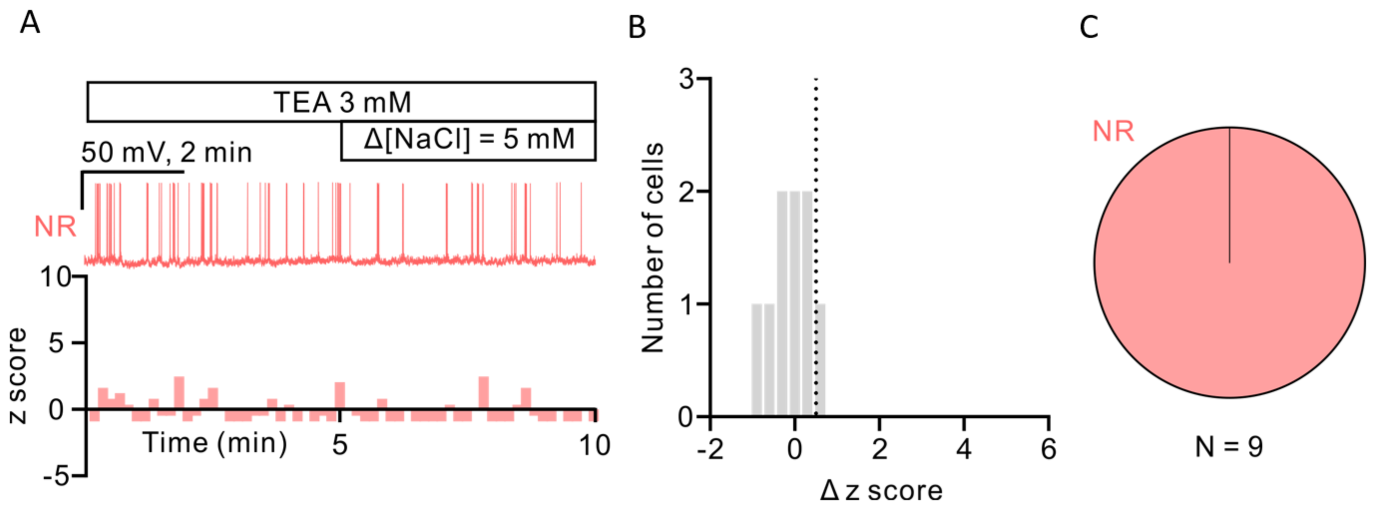

Supplementary Figure 14. Pharmacological inhibition of Kv3.1 abolishes hypertonicity-induced spike generation in PVN-projecting OVLT neurons. (**A**) Top, a representative trace of spontaneous firing recorded from a NaCl-NR neuron after TEA treatment. Bottom, histogram of z score from a representative NaCl-NR cell. (**B**) Distribution of the Δz score in response to 5 mM NaCl stimulation of all TEA-treated cells. Dashed line indicates 0.5. (**C**) Pie chart showing distribution of TEA-treated NaCl-R and NaCl-NR PVN-projecting OVLT neurons. 9 out of 9 TEA-treated neurons are non-responsive to hypertonicity stimulation.
